# A novel anoikis-related gene signature predicts prognosis in patients with sepsis and reveals immune infiltration

**DOI:** 10.1038/s41598-024-52742-9

**Published:** 2024-01-28

**Authors:** Yonghua Wang, Yanqi Chi, Cheng Zhu, Yuxuan Zhang, Ke Li, Jiajia Chen, Xiying Jiang, Kejie Chen, Shuping Li

**Affiliations:** 1 Department of Emergency, The First Affiliated Hospital of Chengdu Medical College, Chengdu, 610500 Sichuan People’s Republic of China; 2 School of Public Health, Chengdu Medical College, Chengdu, 610500 Sichuan People’s Republic of China; 3 Department of Critical Care Medicine, The First Affiliated Hospital of Chengdu Medical College, Chengdu, 610500 Sichuan People’s Republic of China

**Keywords:** Computational biology and bioinformatics, Drug discovery, Immunology, Biomarkers

## Abstract

Sepsis is a common acute and severe medical condition with a high mortality rate. Anoikis, an emerging form of cell death, plays a significant role in various diseases. However, the role of anoikis in sepsis remains poorly understood. Based on the datasets from Gene Expression Omnibus and anoikis-related genes from GeneCards, the differentially expressed anoikis-related genes (DEARGs) were identified. Based on hub genes of DEARGs, a novel prognostic risk model was constructed, and the pattern of immune infiltration was investigated by CIBERSORT algorithm. And small molecule compounds targeting anoikis in sepsis were analyzed using Autodock. Of 23 DEARGs, CXCL8, CFLAR, FASLG and TP53 were significantly associated with the prognosis of sepsis (*P* < 0.05). Based on the prognostic risk model constructed with these four genes, high-risk population of septic patients had significant lower survival probability than low-risk population (HR = 3.30, *P* < 0.001). And the level of CFLAR was significantly correlated with the number of neutrophils in septic patients (r = 0.54, *P* < 0.001). Moreover, tozasertib had low binding energy with CXCL8, CFLAR, FASLG and TP53, and would be a potential compound for sepsis. Conclusively, our results identified a new prognostic model and potential therapeutic molecular for sepsis, providing new insights on mechanism and treatment of sepsis.

## Introduction

Sepsis, a syndrome caused by infection, is a frequently encountered acute and severe medical condition, and, annually, there are approximately 50.9 million cases of sepsis reported worldwide, with a mortality rate of approximately 16%^1,2^. Though the pathogenesis of sepsis is extremely complex, numerous literatures indicated that various pathophysiological processes, including inflammation imbalance, immune dysfunction, dysregulation of apoptosis and autophagy, ultimately lead to organ dysfunction in septic patients^3^. Among these pathogenesis, dysfunction of immune cells, like dendritic cell, monocyte/macrophage, natural killer (NK) cell and lymphocyte, are highlighted in the development of sepsis^4–7^. And Liu et al. reported that the immune cell counts could serve as independent predictors of 28-day mortality in septic patients^8^.

Anoikis is a distinct form of apoptosis initiated by loss of cell adhesions from the extracellular matrix or inappropriate cell adhesion^9^, and occurs in malignancies, cardiovascular disease, diabetes and infections. As an important trigger of sepsis, infection is significantly involved with anoikis in various conditions. Hepatitis B virus suppressed anoikis in HBV-associated liver cancer^10^, *Stenotrophomonas maltophilia* and pathogenic *Pseudomonas aeruginosa* induced anoikis of human lung epithelial cells and vascular cells^11,12^, respectively.

Therefore, dysregulated anoikis could play a role in the development of sepsis. Although there are a few literatures reporting that the anoikis of immune cell could be affected by cadmium exposure^13^ and low density lipoproteins stimulation^14^, the effect of anoikis in sepsis remains unknown. In the present study, based on the data of sepsis patients from Gene Expression Omnibus and anoikis-related genes (ARGs) from GeneCards, comprehensive and multiscale analyses were performed to identify the differentially expressed anoikis-related genes (DEARGs) and essential prognostic ARGs in sepsis. The Gene Ontology and Kyoto Encyclopedia of Genes and Genomes pathways analyses of DEARGs were used to find potential biological pathways. Then, through univariate analysis, Least absolute shrinkage and selection operator (LASSO) regression and multivariate Cox regression, a new prognostic risk model was constructed for septic patients. Furthermore, we also explored the correlation of immune infiltration and prognostic model, miRNA regulatory network and potential therapeutic compounds. The present investigation identified key ARGs and novel prognostic model of sepsis, which could be significant in the diagnosis and treatment of sepsis.

## Materials and methods

### Data collection

The RNA-seq transcriptome data and clinical information were collected from the GEO database (https://www.ncbi.nlm.nih.gov/gds/). The GSE57065, GSE65682 and GSE28750 datasets used in the present study were based on GPL570, GPL13667 and GPL570 platforms, respectively. 254 anoikis-related genes (ARGs) (Supplementary Table 1) were obtained similarly with a previous report^15^. Briefly, ARGs were collected from the GeneCards database (https://www.genecards.org/), and genes with a relevance score > 1.5 were included in the present study.

### Identification of differentially expressed anoikis-related genes (DEARGs)

Based on the GSE57065 dataset, the differentially expressed genes (DEGs) (*P* < 0.05 and |log FC|> 1.5) between the septic patients (n = 82) and healthy controls (n = 25) were analyzed using “limma” R package. Then, based on DEGs and ARGs, DEARGs were screened by Venn diagram using an online tool (http://bioinformatics.psb.ugent.be/webtools/Venn). And the “ggplot2” and “pheatmap” R packages were used for visualization of heatmaps and volcano maps of DEGs and DEARGs. The DEARGs from GSE57065 were validated in GSE28750 dataset.

### Hub genes and potential biological functions of DEARGs in sepsis

A PPI network was established using String databases to evaluate the interactions among DEARGs. According to the previous study^16^, the identification of hub genes of DEARGs was achieved by utilizing MCC algorithm of the CytoHubba plug-in within Cytoscape software (version 3.9.1). And gene ontology (GO) and Kyoto Encyclopedia of Genes and Genomes (KEGG)^17^ pathway analyses were executed using the R software clusterProfiler (version 3.14.3) to extract the outcomes of gene set enrichment.

### Relationship between immune infiltration and sepsis

The “ggplot2” R package was used to compare the abundance of immune cell infiltration between septic patients and healthy controls, and to visually analyze the patterns of immune infiltration. The correlation between immune infiltration patterns and sepsis were performed by CIBERSORT algorithm.

### Identification of anoikis subtypes in sepsis

To unveil distinct anoikis subtypes in sepsis, unsupervised clustering analysis of DEARGs was constructed utilizing the “Consens-usClusterPlus” R package^18^. Our approach employed clustering based on a 1-Spearman correlation distance and was iterated ten times, using 80% of the samples each time. The optimal number of clusters was determined through an empirical cumulative distribution function plot. Principal Component Analysis (PCA) was executed using the R package “ggplot2”.

Then, the immune infiltration patterns among distinct subtypes of anoikis were examined by computing the scores of 22 immune cells through CIBERSORTx^19^. The “ggplot2” R package was used to compare the abundance of immune cell infiltration in two subtypes of anoikis, and to visually analyze the immune cell infiltration.

### Prognostic risk model of DEARGs in sepsis

GSE65682 database, including peripheral blood sequencing data of 479 septic patients and 42 healthy controls were divided into training group and validation group. Within the training group, the genes (*P* < 0.2 in univariate analysis) were included in LASSO Cox and multivariate regression analyses, similar with the previous study^20^. The genes significantly associated with prognosis were prognostic DEARGs and extracted to construct the risk model. The formula for the risk model was as following:

$$\begin{aligned} {\text{Risk Model }} & = \left[ {\left( {{\text{gene 1 expression value }} \times {\text{ b1}}} \right) \, + \left( {{\text{gene 2 expression value }} \times {\text{ b2}}} \right)} \right. \\ & \quad \left. { + \cdots \, + \left( {{\text{gene n expression value }} \times {\text{ bn}}} \right)} \right] \\ \end{aligned}$$

(b is the regression coefficient of the corresponding gene).

And the risk model was validated in the validation group of GSE65682.

### Differences of high- and low-risk population in sepsis

Participants were segregated into high- and low-risk groups based on their median risk scores. Survival analysis was performed by utilizing the "Survival" R package to assess differences between high- and low-risk groups. To evaluate the prognostic effectiveness of the model, we used ROC analysis by the R software package pROC (version 1.17.0.1) to calculate the area under the curve (AUC). And Cox regression analysis was employed to assess the risk model for its independence as a factor in sepsis.

To assess the disparities in immune infiltration between the high and low-risk groups, we investigated immune-related functions using the CIBERSORTx in the study.

### Functions and pathways of DEARGs of high- and low-risk population in sepsis

GeneMANIA (http://www.genemania.org) was used to analyze the relevant enrichment pathways online. Gene Set Variation Analysis (GSVA) employs unsupervised classification of samples through gene expression and the integration of multiple pathway information to discern alterations in pathway activity. We used gene expression profiling as the report of Hanzelmann et al.^21^, and from the Molecular Signatures Database (http://www.gsea-msigdb.org/gsea/downloads.jsp) to download the c2. Cp. Kegg. Subset, using “GSVA” and “limma” R packages to analyze alterations in different pathways and biological functions.

### Construction of miRNA regulation network

The miRNAs related to the prognostic DEARGs in sepsis were predicted by miRNet (https://www.mirnet.ca/). The differentially expressed miRNAs (|log_2_ FC|> 1 and *P* < 0.05) between septic patients and healthy controls in GSE134358 (platform: GPL21572) from GEO were analyzed using “limma” R packages. By overlapping predicted miRNAs of prognostic ARGs with differentially expressed miRNAs, we constructed the miRNA regulatory networks of prognostic DEARGs. Cytoscape software (version 3.9.1) was used for visualization.

### Predict of potential drugs for sepsis

Cmap (https://clue.io/) was used to predict small molecule compounds of prognostic ARGs, and 9 compounds with highest scores were used for further study. The 2D chemical structures of ligands of 9 compounds were downloaded from PubChem (https://pubchem.ncbi.nlm.nih.gov). The PDB format of proteins associated with prognostic genes was then obtained from the PDB database (https://www.rcsb.org/pdb). Protein crystal structures were introduced into Pymol software for dehydration, and subsequently, conduction crystals were introduced into AutoDockTools to construct docking grid boxes for the target. Molecular docking was achieved by AutoDock Vina. And the complexes of proteins and compounds were visualized by Pymol software.

### Statistical analysis

In the present study, all statistical analysis was conducted by R 4.1.1. The Wilcoxon test and the Kruskal–Wallis test were used for comparisons between two independent samples and comparisons among multiple samples for nonparametric data, respectively. The t test or one-way ANOVA were used for parametric data. R packages used in the present study were downloaded from Bioconductor packages or R packages. For each analysis, *P* value < 0.05 was considered statistically significant.

## Results

### Identification of differentially expressed anoikis-related genes (DEARGs) between septic patients and healthy controls

**Figure 1:**
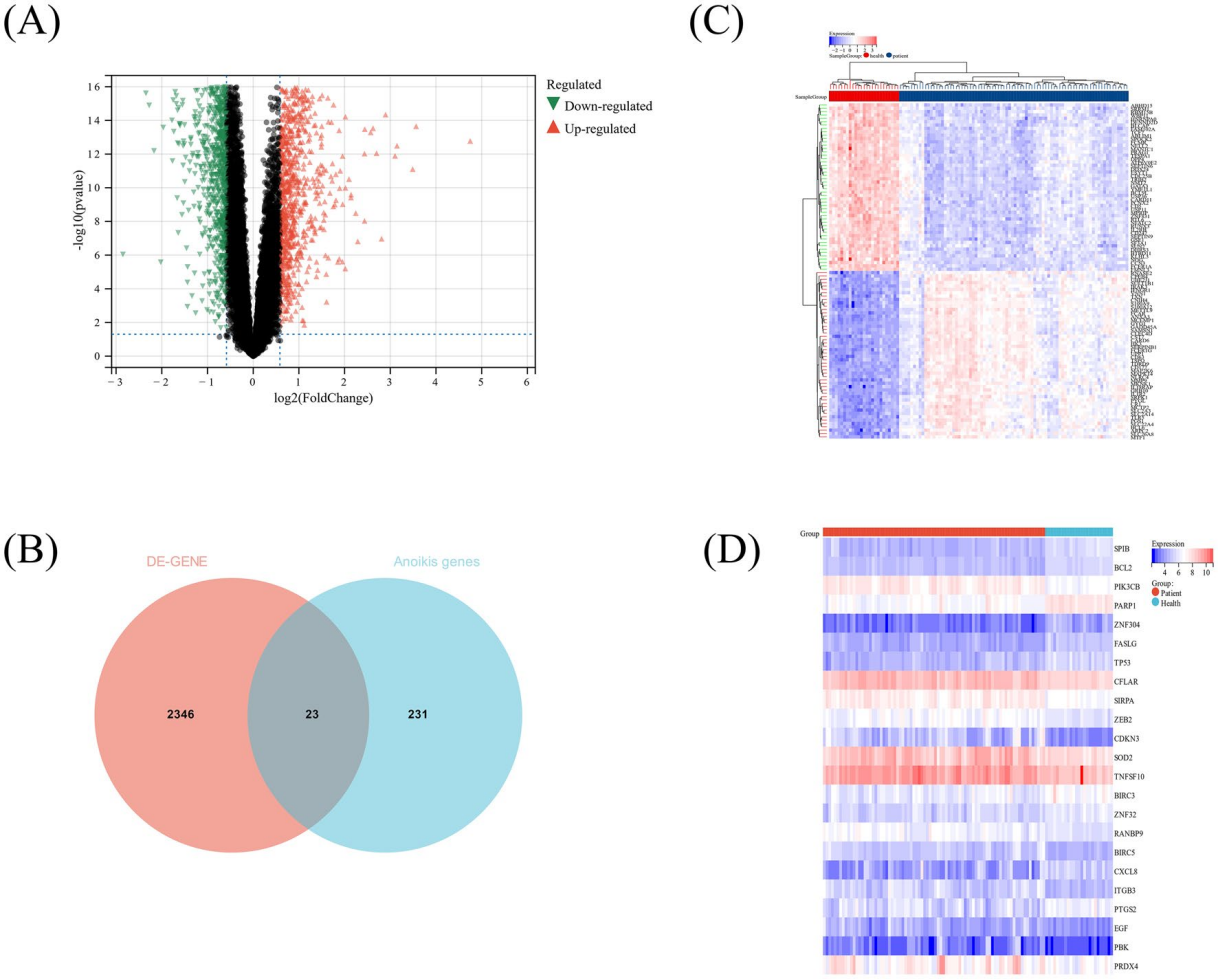
Differentially expressed genes (DEGs) and anoikis-related genes (ARGs) between septic patients and healthy controls. (**A**) Volcano plot of the DEGs. (**B**) Venn Diagram of DEGs and ARGs. (**C**) Heatmap of the DEGs. (**D**) Heatmap of the DEARGs.

By conducting differential analysis, we identified a total of 2369 differentially expressed genes (DEGs) between septic patients and healthy controls within the GSE57065 dataset (*P* < 0.05). The expression patterns of these DEGs are visually depicted utilizing both a heatmap (Fig. 1A) and a volcano plot (Fig. 1C). Of these DEGs, there were 23 DEARGs (Fig. 1B), and the expression patterns of these DEARGs were visualized as a heatmap (Fig. 1D). The expressions of DEARGs of septic patients and healthy controls were shown in Supplementary Fig. 1.

### Protein–protein interaction (PPI) network and functional analysis of DEARGs

**Figure 2:**
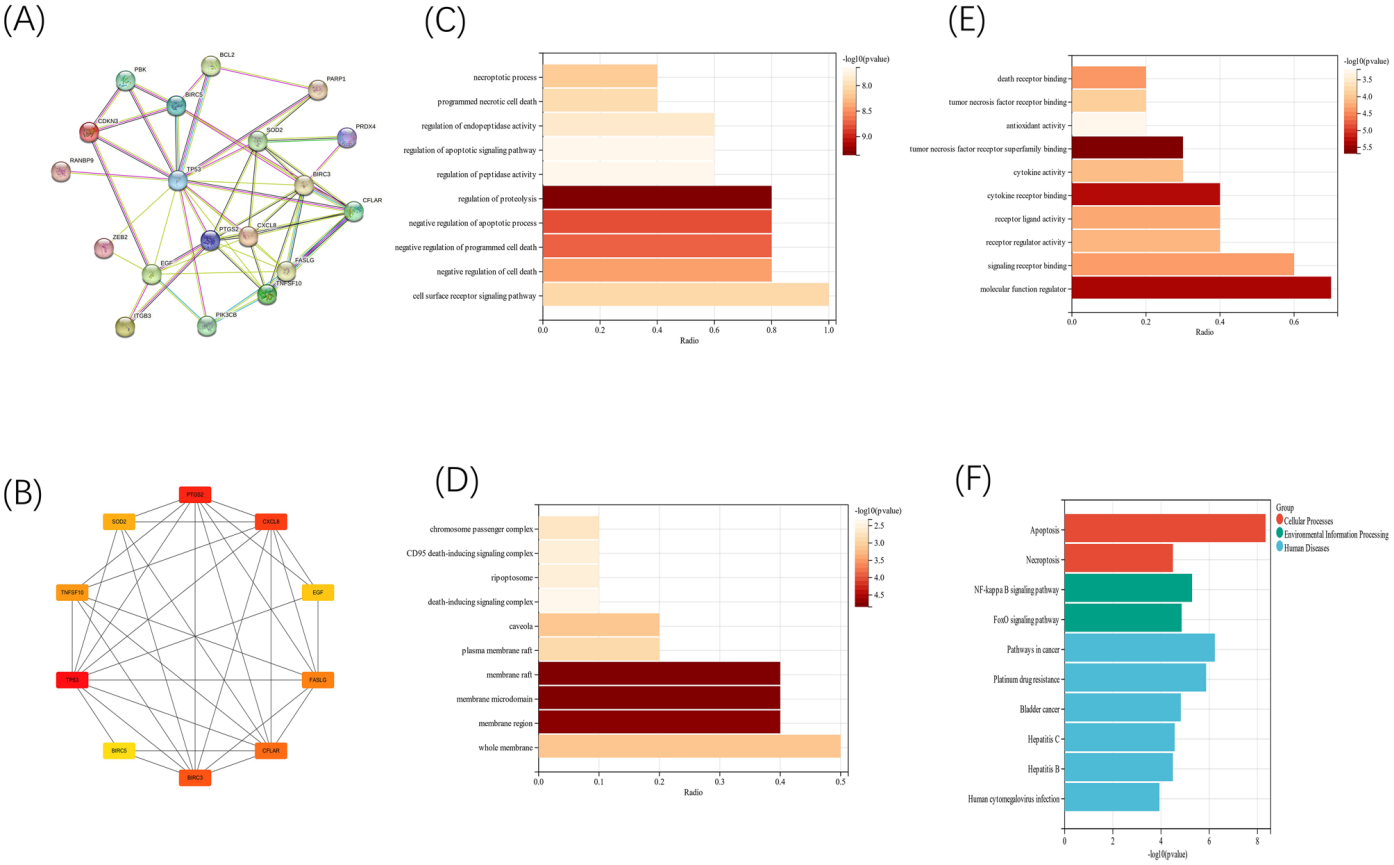
PPI network and functional analysis of DEARGs. (**A**) PPI network of the DEARGs. (**B**) Hub genes identified by CytoHubba (Top 10). (**C**) Biological processes of GO analysis of DEARGs (Top 10). (**D**) Cellular components of GO analysis of DEARGs (Top 10). (**E**) Molecular function of GO analysis of DEARGs (Top 10). (**F**) KEGG pathway analysis of DEARGs (Top 10).

Among these DEARGs, using PPI network, 10 hub genes were identified, including TP53, PTGS2, CXCL8, BIRC3, CFLAR, FASLG, TNFSF10, SOD2, EGF and BIRC5, and TP53 had the highest score (Fig. 2A,B). To ascertain the biological functions and signaling pathways of the DEARGs, GO and KEGG pathway analyses were performed. Notably, the GO analysis revealed that the hub genes of DEARGs in septic patients were mainly from membrane and death-inducing signaling complex, and functioning in tumor necrosis factor superfamily binding (Fig. 2C–E). Through KEGG pathway analysis, various pathways of the DEARGs in sepsis patients were highlighted, including apoptosis, necroptosis, NF-κB and FoxO pathways and infection (Fig. 2F).

### Relationship between DEARGs and immune cells

**Figure 3:**
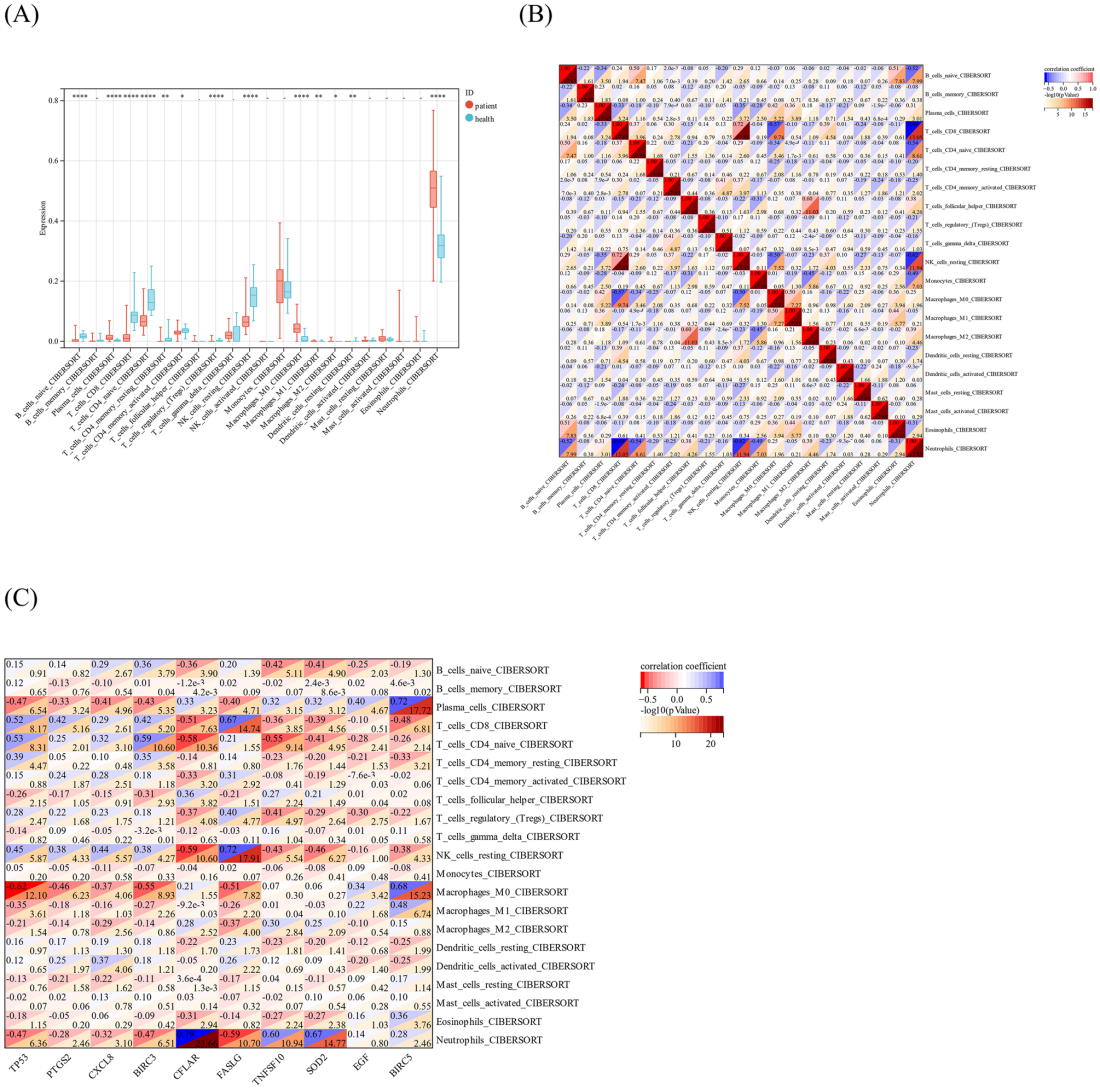
Immune cell infiltration and DEARGs in sepsis. (**A**) Components of immune cells in the septic patients and healthy controls. (**B**) Correlation of immune cells. (**C**) Correlations of hub genes of DEARGs and immune cells.

To investigate the association between DEARGs and immune cells, the variations in the abundance of infiltrating immune cells in each sample were studied. The data showed that various types of immune cells were significantly different in the septic patients when compared with the healthy controls (*P* < 0.05), including up-regulated (neutrophil, M0 macrophage, plasma cell and) and down-regulated cells (naïve B cell, CD8 T cell, naïve CD4 T cell, resting/activated CD4 memory T cell, and resting NK cell). The heatmap of the correlation between immune cells showed that there was a significant negative correlation between neutrophils and monocytes and eosinophils, while there was a significant positive correlation between eosinophils and monocytes (Fig. 3A,B). Correlation analysis revealed strong associations between DEARGs and specific immune cell types, including naïve B cell, eosinophil, M0 macrophage, M2 macrophage, neutrophils, activated memory CD4 T cell, plasma cell, and regulatory T cells (Tregs) (correlation coefficient > 0.60, *P* < 0.05) (Fig. 3C). These results indicated that the DEARGs could contribute to the changed immune environment in sepsis.

### Point clusters associated with anoikis in sepsis

**Figure 4:**
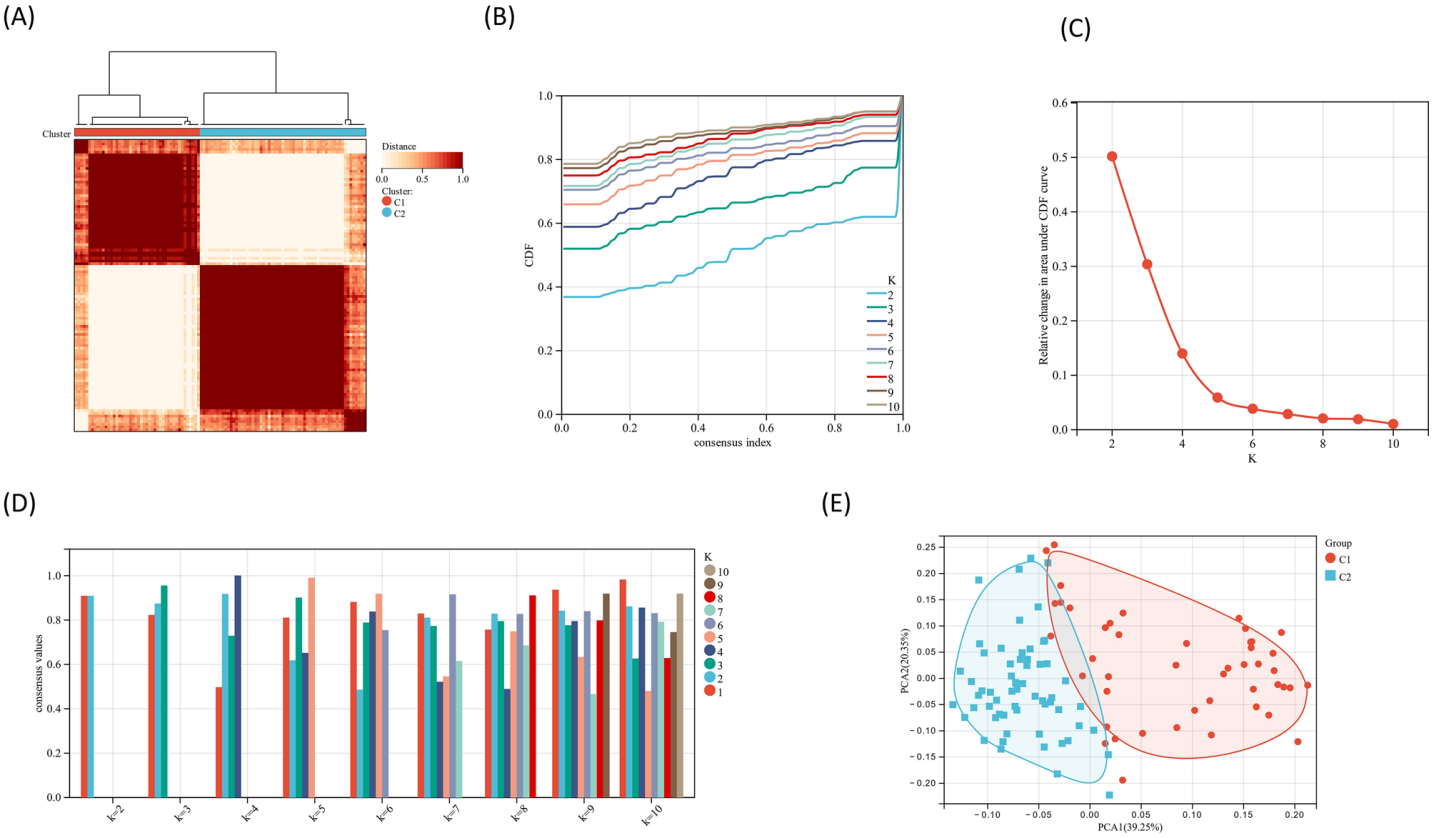
Identification of molecular clusters associated with anoikis in sepsis. (**A**) Consensus clustering matrix (K = 2). (**B**) Cumulative distribution function (CDF) curves (K = 2–9, respectively). (**C**) Representative CDF delta area curves. (**D**) Consensus clustering scores (K = 2–9, respectively). (**E**) Two clusters by PCA.

To gain deeper insights into the classification of anoikis-related patterns, we applied unsupervised consensus cluster analysis to categorize 69 sepsis samples. Interestingly, when setting K = 2, the consensus index within the cumulative distribution function (CDF) curve exhibited minimal fluctuations. Consequently, septic patients were divided into two clusters, C1 (n = 46) and C2 (n = 61) (Fig. 4A–D). Through principal components analysis (PCA), C1 was significantly differed from C2 (Fig. 4E).

### Immune microenvironment and biological function of different subgroups of anoikis

**Figure 5:**
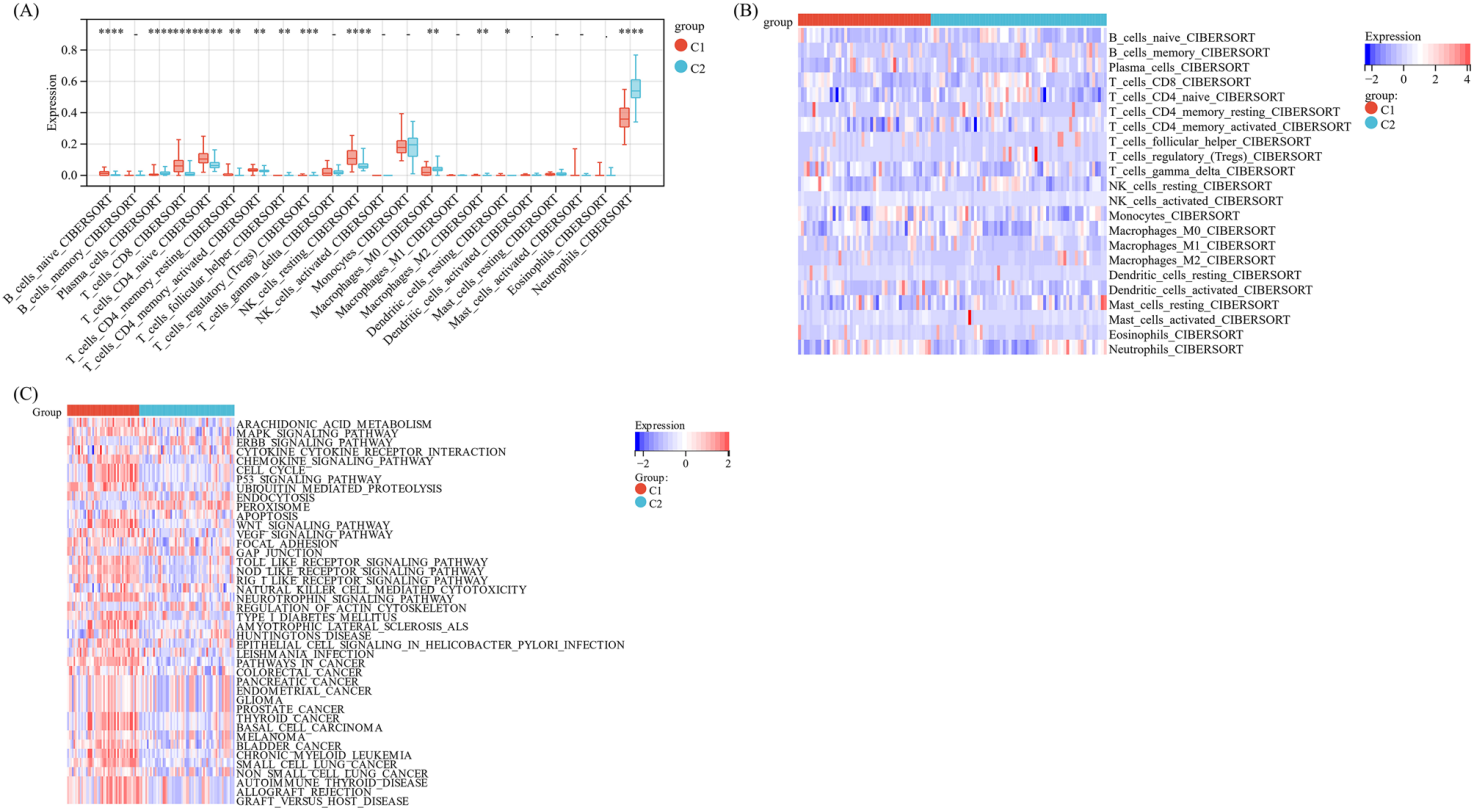
Characterizations of infiltration of immune cells in different subtypes of sepsis. (**A**) Components of immune cells in the subtypes of sepsis (**B**) Heatmap of immune cells in the subtypes of sepsis. (**C**) Heatmap of GSVA in the subtypes of sepsis.

To examine the disparities of infiltrated immune cells between the different anoikis subtypes, we meticulously analyzed the immune infiltration patterns of each subtype. The findings demonstrated that, compared with the Cluster 2, expressions of naïve B cell, CD8 T cell, naïve CD4 T cell, resting/activated memory CD4 T cell and resting NK cell were significantly increased in the Cluster 1 (*P* < 0.05), and the levels of plasma cell, M0 macrophage and neutrophil were decreased (*P* < 0.05) (Fig. 5A,B). This observation underscores the distinct immune infiltration characteristics between the two subtypes of anoikis in sepsis. And the data of GSVA analysis on the distinctive genes associated with these subtypes showed that Cluster C1 was prominently related to peroxidase, P53 signaling, apoptosis and Toll-like receptor pathways, while Cluster C2 was notably associated with ERBB signaling pathway and actin regulatory pathways (Fig. 5C).

### Prognostic risk model of DEARGs in sepsis

**Figure 6:**
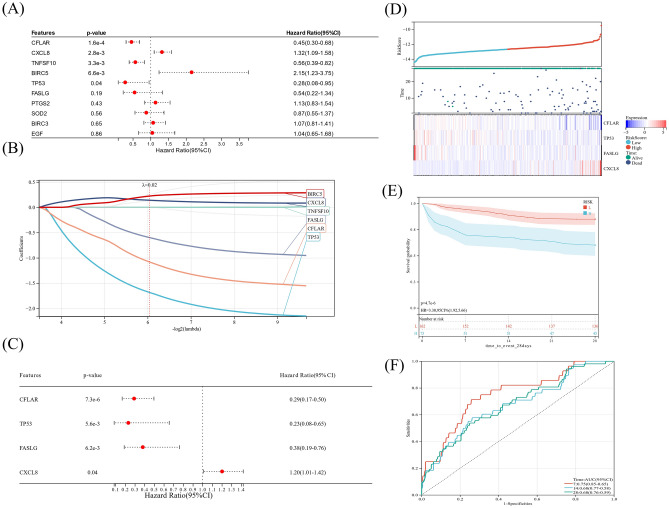
Prognostic risk score model of DEARGs in the patients with sepsis. (**A**) Univariate analysis of hub DEARGs in sepsis. (**B**) LASSO Cox regression of hub DEARGs in sepsis. (**C**) Multivariate Cox regression of hub DEARGs in sepsis. (**D**) Patient distribution and survival time based on risk score. (**E**) Kaplan–Meier curves for the survival of septic patients in low- (L) and high-risk (H) groups. (**F**) ROC curves of septic patients with 7-, 14- and 28-day survival.

As shown in Fig. 6A–C, univariate analysis identified 6 genes (CXCL8, CFLAR, FASLG, TP53, BIRC5, TNFSF10) as prognostic factors (*P* < 0.2). And the data of LASSO-Cox and multivariate regression indicated that the prognostic DEARGs included CXCL8, CFLAR, FASLG and TP53. The formula was derived as following:

$$\begin{aligned} {\text{Prognostic Risk Model}} & = - \;{1}.{17}0{2799}0{8}\; \times \;{\text{CFLAR}} - {1}.{37242248}\; \times \;{\text{TP53}} - 0.{9}0{9225}00{2}\; \times \;{\text{FASLG}} \\ & \quad + 0.{154752771}\; \times \;{\text{CXCL8}} \\ \end{aligned}$$

Notably, CXCL8 demonstrated a positive association with increased risk (HR > 1), while CFLAR and TP53, along with FASLG, exhibited protective factors with HR < 1. Based on the median risk score, the cohort of 240 septic patients was stratified into two distinct categories: high-risk group encompassing 120 individuals and the low-risk group consisting of 120 patients. The results suggested that the 28-day survival rate of the septic patients in the low-risk group was significantly lower than that in the high-risk group (*P* < 0.05) (Fig. 6D,E). The AUC values of ROC curves of septic patients with 7-, 14- and 28-day survival were more than 0.65, and highest AUC value was observed at 7 day (Fig. 6F).

**Figure 7:**
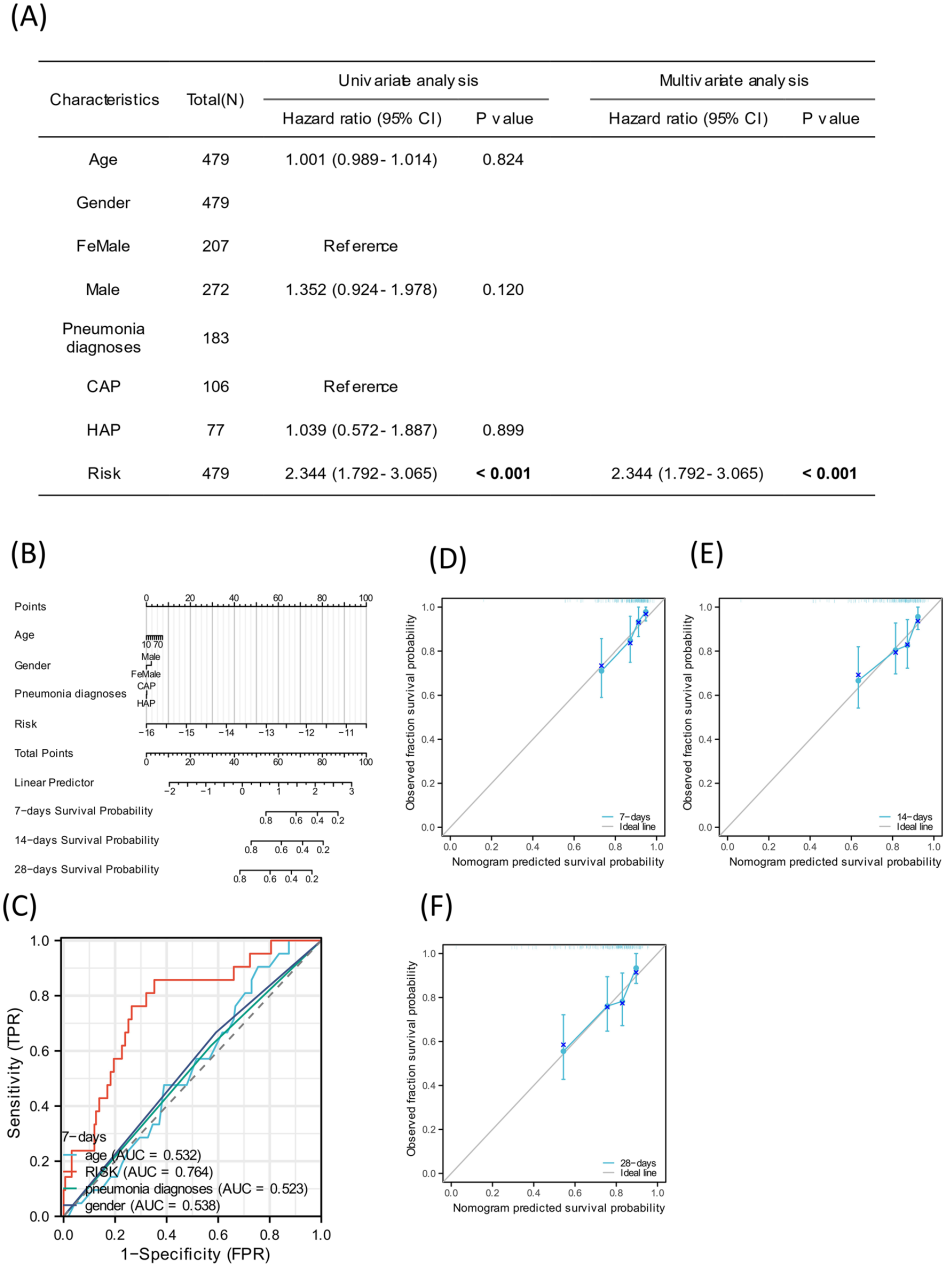
Prognostic value of risk score in sepsis. (**A**) Univariate analysis of the characteristics and risk score of septic patients. (**B**–**C**) Prognostic nomogram of overall survival and ROC curve. (**D**–**F**) Calibration curve of the nomogram for predicting 7-, 14-, and 28-day survival of septic patients.

To create a prognostic nomogram, we combined the risk score of overall survival with various clinical risk factors. According to the results of multivariate regression analysis and nomogram, it indicated that the risk score was an independent prognostic indicator (Fig. 7A–C). This calibration curve indicates the predicted probability and actual probability (Fig. 7D–F).

### Validation of prognostic risk score model

**Figure 8:**
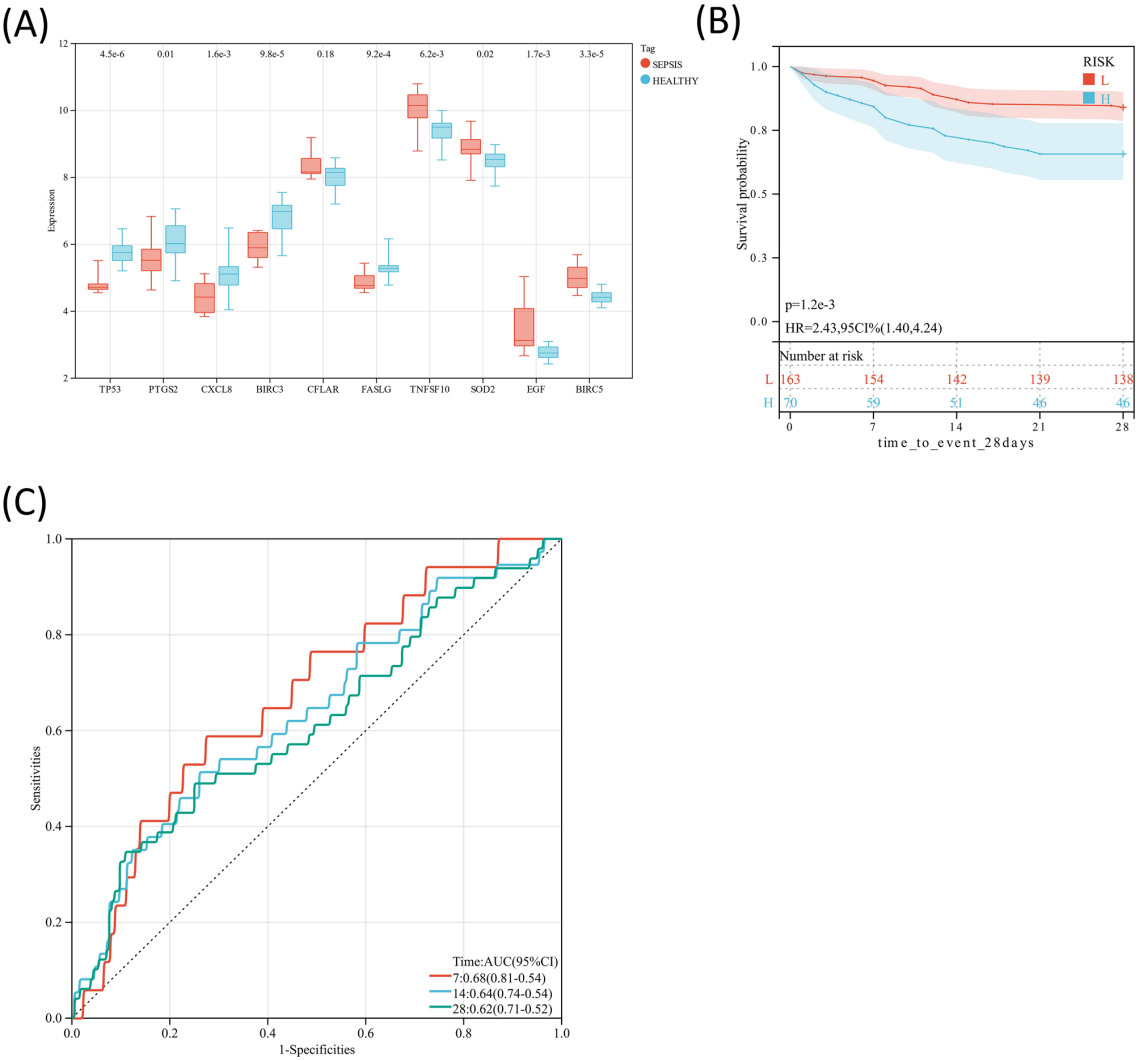
Validation of prognostic risk score model. (**A**) Expression of hub genes of DEARGs between septic patients and healthy controls. (**B**) Kaplan–Meier curves for the survival of septic patients in low- (L) and high-risk (H) groups. (**C**) ROC curves of septic patients with 7-, 14- and 28-day survival.

In the validation dataset (GSE58294), the 10 hub genes of DEARGs, except CFLAR, were significantly different between the septic patients and healthy controls (*P* < 0.05) (Fig. 8A), similar with the results obtained from GSE57065 dataset. And the 28-day survival rate of the septic patients from the low-risk group was significantly higher than that from the high-risk group (*P* < 0.05) (Fig. 8B). Upon validation through GSE58294 dataset, the survival outcomes distinctly favored the low-risk group over the high-risk group (Fig. 8C).

### Difference of immune cells between low- and high-risk groups in sepsis

**Figure 9:**
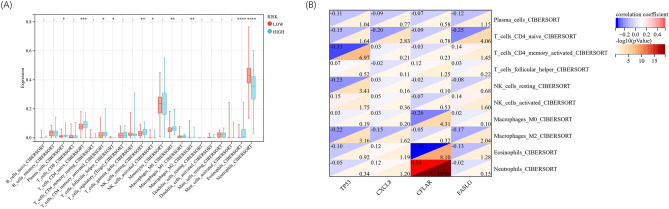
Analysis of immune infiltration in low- and high-risk population of septic patients. (**A**) Patterns of immune infiltration. (**B**) Correlations of ARGs and immune cells.

Compared with the high-risk group, the levels of M2 macrophage and neutrophil were markedly increased in the low-risk group (*P* < 0.05), and the expressions of plasma cell, naïve CD4 T cell, activated memory CD4 T cell, follicular helper T cell, resting NK cell, M0 macrophage and eosinophil were significantly decreased (*P* < 0.05) (Fig. 9A). The correlation analysis indicated significant associations between DEARGs and immune cells, including CFLAR and neutrophil, CFLAR and eosinophil, and TP53 and activated memory CD4 T cell (Fig. 9B).

### Functions of prognostic DEARGs and pathways between low- and high-risk patients with sepsis

**Figure 10:**
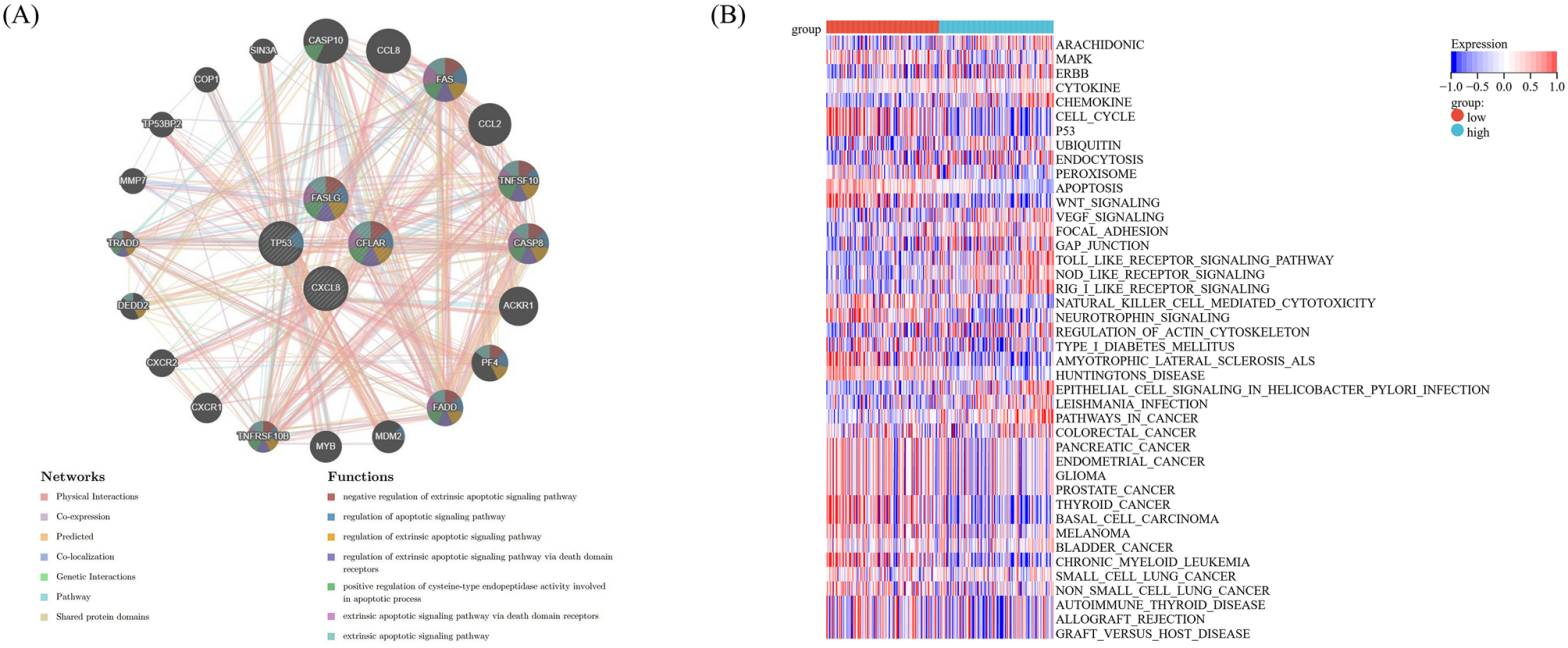
Targets of prognostic DEARGs and pathways between low- and high-risk patients with sepsis. (**A**) Interaction network of prognostic DEARGs by GeneMANIA analysis. (**B**) Heatmap of low- and high-risk group of septic patients.

Based on the GeneMANIA analysis, four prognostic DEARGs were involved with progression of apoptosis by regulating various targets, like CASP8, FAS and FADD (Fig. 10A). The data of GSVA showed that there were 42 pathways statistically significant between the low- and high-risk population, mainly MAPK pathway, TOLL signal pathway, cytokine receptor interaction, chemokine signaling pathway and P53 pathway. The high-risk group exhibited associations with NOD-like receptors and MAPK receptors (Fig. 10B).

### Regulation network of miRNA-mRNA

**Figure 11:**
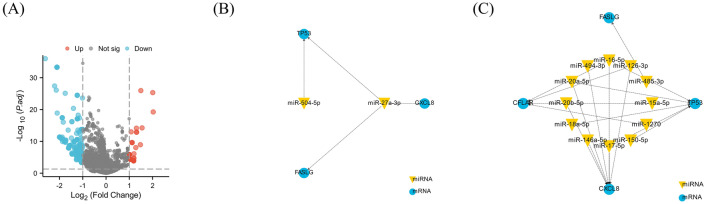
miRNA regulatory network of prognostic DEARGs. (**A**) Volcano plot of differentially expressed miRNAs between septic patients and healthy controls. (**B**) Regulatory network of up-regulated miRNAs. (**C**) Regulatory network of down-regulated miRNAs.

Gene expression is modulated by miRNAs. Using miRNet database, a total of 335 miRNAs were predicted to potentially regulate the expression of prognostic ARGs, 29 miRNAs were found to be targeting to multiple genes (Supplementary Table 2). In GSE134358, there were 324 differentially expressed miRNAs, of which 288 were down-regulated and 36 up-regulated (Fig. 11A and Supplementary Table 3). And differentially expressed miRNAs could regulate the expressions of prognostic DEARGs by binding to mRNAs (Fig. 11B,C).

### Tozasertib could be a potential therapeutic agent for sepsis

**Figure 12:**
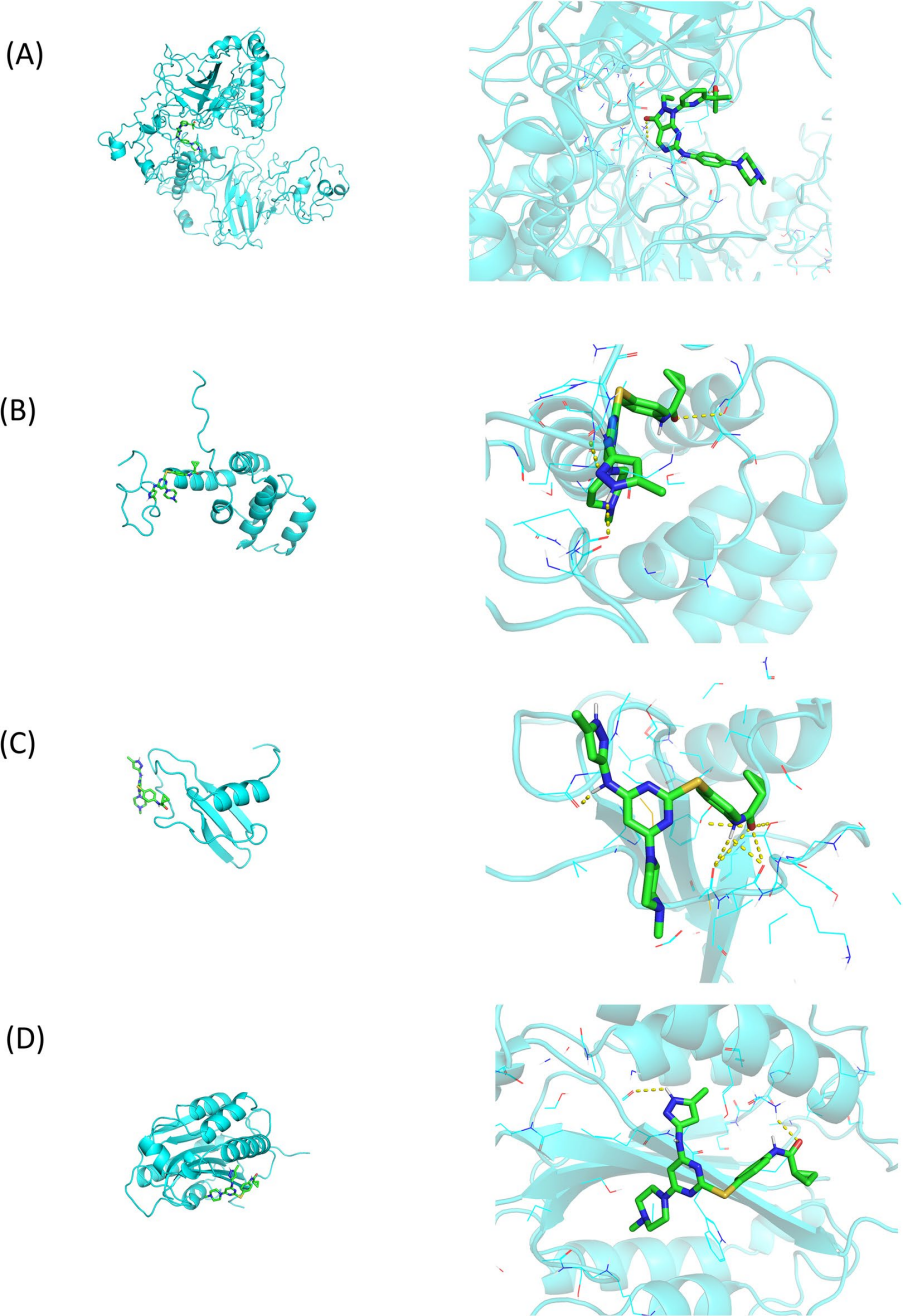
Interactions between (**A**) TP53, (**B**) FASLG, (**C**) CXCL8, (**D**) CFLAR and tozasertib. Hydrogen bonds were displayed as yellow dashed lines.

Through Cmap analysis, several small molecule compounds related to DEARGs in sepsis were identified. The top nine compounds with the highest scores were shown in Table 1, and 2D chemical structures of these compounds were displayed in Supplementary Fig. 2. Subsequently, we employed AutoDock Vina to perform molecular docking of these compounds with the four prognostic DERAGs (Table 2). It was shown that the binding energy was less than 5.5 kcal/mol, indicating that the compound was in the active state. Tozasertib demonstrated binding energies of − 8, − 7.5, − 6.5, and − 7.5 kcal/mol, respectively, indicating a favorable and robust interaction. Tozasertib establishes interactions with the four targets by forming hydrogen bonds at specific sites including SER-33, PHE311, ASF210, CYS-9, ILE10, TYR463, and ASN-447 within proximity to the active site, thereby mediating its biological effects (Fig. 12).

**Table 1:** Small molecule compounds related to prognostic DEARGs.

Compounds	Description	Score
Flubendazole	Tubulin inhibitor	97.43
Flucytosine	Antifungal	97.1
Adavosertib	WEE1 kinase inhibitor	97.04
Tozasertib	Aurora kinase inhibitor	96.86
7-Hydroxyflavone	Antineoplastic	96.58
1-Phenylbiguanide	Serotonin receptor agonist	96.53
SB-203186	Serotonin receptor antagonist	96.51
Mepireserpate	Catecholamine depleting sympatholytic	96.14
Fenbendazole	Tubulin inhibitor	94.64

**Table 2:** Binding energy of compounds with prognostic DEARGs.

Compounds	TP53	FASLG	CXCL8	CFLAR
Flubendazole	− 7.7	− 6.2	− 6.6	− 6.9
Flucytosine	− 5	− 4.9	− 4.4	− 4.9
Adavosertib	− 7.7	− 7.2	− 7.3	− 7
Tozasertib	− 8	− 7.5	− 6.5	− 7.5
7-Hydroxyflavone	− 8.1	− 6.8	− 6.1	− 6.7
1-Phenylbiguanide	− 6.8	− 5.6	− 5.4	− 6.2
SB-203186	− 6.5	− 5.8	− 5.3	− 6.2
Mepireserpate	− 7.7	− 6.7	− 6.4	− 7.2
Fenbendazole	− 6.3	− 6.2	− 5.9	− 6.7

## Discussion

Over an extended period, researchers have dedicated their efforts to unraveling the intricate mechanisms and treatment strategies for sepsis, with the aim of enhancing patient prognosis. Previous investigations have established a clear link between sepsis and immune development^2,22^, leading to the identification of several biomarkers for prognosis assessment^23^. However, the inherent complexity of pathological mechanisms of sepsis has posed a challenge in pinpointing a singular biomarker capable of accurately predicting its prognosis^24^. Consequently, there is an urgent need to uncover novel mechanisms and explore innovative biomarkers and treatments for sepsis.

In the present study, we found that, by GO and KEGG pathway analyses, the hub genes of ARGs in sepsis were involved with NF-κB and FoxO pathways, and infection, which was consisted with the triggers and development of sepsis^25,26^. Moreover, DEARGs were significantly associated with the immune infiltration patterns. In line with prior researches^22,27,28^, our data indicated that DEARGs in sepsis exhibited predominant positive correlations with macrophages, neutrophils, and T cells, while displaying a negative correlation with CD4/CD8 ratio of T cells. The immune infiltration patterns were significantly different between subtypes of anoikis in sepsis. Subtype C1 showed elevated expression levels in naïve B cells, CD8 T cells, resting memory CD4 T cells, active CD4 T cells, and resting NK cells. And the subtype C2 had increased expression in plasma cells, macrophage M0 cells, and neutrophils. These data suggested that anoikis was involved with the dysregulation of immune cell and development of sepsis.

To investigate the prognostic significance of DEARGs in sepsis, we firstly constructed a DEARGs-related prognostic model using four DEARGs (CFLAR, TP53, FASLG, CXCL8). CFLAR plays a pivotal role as a regulator of innate immune molecules within the body. Several researched have elucidated that CFLAR is associated with immune regulation, cardiac remodeling, and acute liver injury^29–31^. In the context of sepsis, CFLAR has been identified on neutrophils^32^, and it has been shown to down-regulate lipopolysaccharide-induced NF-κB activation^33^.

TP53 gene mutations and the dysregulation of the TP53 pathway have been recognized as significant contributors to the pathogenesis of inflammatory processes^34^. Within the realm of inflammatory responses, pro-inflammatory macrophages have the capacity to inhibit P53 activity, thus promoting inflammation^34^. Notably, our research indicates a substantial decrease in TP53 levels among sepsis patients, suggesting that higher TP53 level might potentially exert a positive effect in sepsis. And the precise mechanism by which TP53 operates in sepsis remains elusive, and our study may offer fresh insights into unraveling this intricate mechanism.

FASLG, a member of the tumor necrosis factor receptor/nerve growth factor receptor superfamily, is prominently expressed on the surfaces of diverse cell types, with a notable presence on various immune cells. Its involvement spans immunity and inflammation^35^. It has been reported that FASLG mediates apoptosis in severe sepsis^36^, and inhibition of FAS/FASLG signaling prevents hepatic injury during sepsis^37^ and improves septic survival^38^.

CXCL8 is a chemokine that holds a critical role as a human neutrophil chemoattractant, especially during responses to infection and tissue damage. In instances of bodily inflammation, CXCL8 engages neutrophils, enabling them to roll on endothelial surfaces. Upon activation by chemical stimulus ligands, subsequent migration is triggered, often leading to damage across multiple organs. CXCL8 also possesses the ability to activate monocytes, CD8 T cells, and mast cells, thereby contributing to the initiation of immune responses^39^. During the onset of sepsis, the neutrophils recognize the chemotactic signals from CXCL8, promoting their migration to sites of infection and contributing to the series of responses associated with sepsis development^40^. In the present study, the new prognostic model based on the ARGs would shed light on sepsis development and potential treatment strategies.

Using the prognostic model, septic patients were divided into high-risk group and low-risk group to further study the different patterns of immune infiltration. Neutrophils and T cells exhibited higher scores within the high-risk group, consistent with prior investigations^22,40^. Several researches have been shown that neutrophil dysfunction stands as a pivotal contributor to multiple organ failure in sepsis, and the compromised migration of neutrophils bears direct implications for sepsis prognosis^40–43^. Moreover, T cell-mediated immune responses also play a crucial role in sepsis. In the immediate aftermath of sepsis onset, CD4 and CD8 T cells often demonstrate a depleted state accompanied by significant functional impairments^44,45^. Our data indicated that anoikis might potentially influence sepsis development and prognosis via impacting immune infiltration.

To search for relevant small molecule drugs, we employed the Cmap database to identify compounds with potential impacts on anoikis in sepsis. Among all compounds, tozasertib exhibited favorable binding energy. Tozasertib, a class of small molecules, functions as an ATP-competitive inhibitor of PLK1/Wee1 kinase^46^. Previous investigations have illuminated its effects on conditions such as tumors, pulmonary hypertension, and type 1 diabetes^47–49^. Our data suggested the potential of tozasertib as a small molecule drug, offering novel therapeutic possibilities for future sepsis treatment once the effect of tozaserib was validated by in vivo and in vitro study.

The present study has two limitations: 1. The data needs to be verified by more samples of septic patients and healthy controls. 2. The effect of tozaserib in sepsis would be further determined by in vivo and in vitro study.

## Conclusion

The present study firstly unveiled a novel role of anoikis in sepsis. The anoikis-related genes and immune infiltration pattern were significantly involved with sepsis. Of differentially expressed anoikis-related genes, CFLAR, TP53, FASLG and CXCL8 were significantly associated with the prognosis of sepsis. Based on these four genes, a new prognostic model was constructed and significantly predicted distinct survival between low- and high-risk patients with sepsis. Moreover, tozasertib would be a potential compound for treatment of sepsis by targeting anoikis. Our findings shed light on the role of anoikis in sepsis, and provided new ways to illustrate pathogenesis and explore treatment strategies of sepsis.

### Supplementary Information

Supplementary Figure 1.

Supplementary Figure 2.

Supplementary Table 1.

Supplementary Table 2.

Supplementary Table 3.

## Data Availability

The datasets presented in this study can be found in online repositories. The names of the repository/repositories and accession numbers can be found in the article. The study of GSE134358 and GSE65682 was approved by institutional review boards of two tertiary teaching hospitals in the Netherlands (Academic Medical Center, Amsterdam and University Medical Center Utrecht, Utrecht). The study of GSE57065 was approved by Comité consultatif de Protection de Personnes (CPP) de Lyon A. The study of GSE28750 was approved by institutional review boards (IRBs)/Human Research Ethics Committees (HRECs) from Mater Health Services (MHS), Uniting Care, the Royal Brisbane & Women's Hospital and the Nepean Hospital Human Research Ethics Committee.

## References

[1] Fleischmann C (2016). Assessment of global incidence and mortality of hospital-treated sepsis. Current estimates and limitations. Am. J. Respir. Crit. Care Med..

[2] Cecconi M, Evans L, Levy M, Rhodes A (2018). Sepsis and septic shock. Lancet.

[3] Huang M, Cai S, Su J (2019). The pathogenesis of sepsis and potential therapeutic targets. Int. J. Mol. Sci..

[4] Efron PA (2004). Characterization of the systemic loss of dendritic cells in murine lymph nodes during polymicrobial sepsis. J. Immunol. (Baltimore, Md.: 1950).

[5] Cheng SC (2016). Broad defects in the energy metabolism of leukocytes underlie immunoparalysis in sepsis. Nat. Immunol..

[6] Guo Y, Patil NK, Luan L, Bohannon JK, Sherwood ER (2018). The biology of natural killer cells during sepsis. Immunology.

[7] Schäfer ST (2016). Mitochondrial DNA: An endogenous trigger for immune paralysis. Anesthesiology.

[8] Liu S, Li Y, She F, Zhao X, Yao Y (2021). Predictive value of immune cell counts and neutrophil-to-lymphocyte ratio for 28-day mortality in patients with sepsis caused by intra-abdominal infection. Burns Trauma.

[9] Frisch SM, Francis H (1994). Disruption of epithelial cell-matrix interactions induces apoptosis. J. Cell Biol..

[10] Tu W, Gong J, Tian D, Wang Z (2019). Hepatitis B virus X protein induces SATB1 expression through activation of ERK and p38MAPK pathways to suppress anoikis. Dig. Dis. Sci..

[11] DuMont AL, Cianciotto NP (2017). Stenotrophomonas maltophilia serine protease StmPr1 induces matrilysis, anoikis, and protease-activated receptor 2 activation in human lung epithelial cells. Infect. Immun..

[12] Beaufort N (2011). The thermolysin-like metalloproteinase and virulence factor LasB from pathogenic Pseudomonas aeruginosa induces anoikis of human vascular cells. Cell. Microbiol..

[13] Guan TQ (2022). Cadmium-induced splenic lymphocytes anoikis is not mitigated by activating Nrf2-mediated antioxidative defense response. J. Inorgan. Biochem..

[14] Escate R, Padro T, Badimon L (2016). LDL accelerates monocyte to macrophage differentiation: Effects on adhesion and anoikis. Atherosclerosis.

[15] Diao X, Guo C (2023). Identification of a novel anoikis-related gene signature to predict prognosis and tumor microenvironment in lung adenocarcinoma. Thorac. Cancer.

[16] Zhang Y (2020). Bioinformatics analysis of potential core genes for glioblastoma. Biosci. Rep..

[17] Kanehisa M, Furumichi M, Sato Y, Kawashima M, Ishiguro-Watanabe M (2023). KEGG for taxonomy-based analysis of pathways and genomes. Nucleic Acids Res..

[18] Wilkerson MD, Hayes DN (2010). ConsensusClusterPlus: A class discovery tool with confidence assessments and item tracking. Bioinformatics.

[19] Newman AM (2015). Robust enumeration of cell subsets from tissue expression profiles. Nat. Methods.

[20] Chandra A, Xing W, Kadhim MR, Williamson TH (2014). Suprachoroidal hemorrhage in pars plana vitrectomy: Risk factors and outcomes over 10 years. Ophthalmology.

[21] Hanzelmann S, Castelo R, Guinney J (2013). GSVA: Gene set variation analysis for microarray and RNA-seq data. BMC Bioinform..

[22] van der Poll T, Shankar-Hari M, Wiersinga WJ (2021). The immunology of sepsis. Immunity.

[23] Faix JD (2013). Biomarkers of sepsis. Crit. Rev. Clin. Lab. Sci..

[24] Sinha M (2018). Emerging technologies for molecular diagnosis of sepsis. Clin. Microbiol. Rev..

[25] Huang S (2022). Tim-3 regulates sepsis-induced immunosuppression by inhibiting the NF-κB signaling pathway in CD4 T cells. Mol. Ther.: J. Am. Soc. Gene Ther..

[26] Smith IJ (2010). Sepsis increases the expression and activity of the transcription factor Forkhead Box O 1 (FOXO1) in skeletal muscle by a glucocorticoid-dependent mechanism. Int. J. Biochem. Cell Biol..

[27] Venet F, Monneret G (2018). Advances in the understanding and treatment of sepsis-induced immunosuppression. Nat. Rev. Nephrol..

[28] Lin CW (2014). T-cell autophagy deficiency increases mortality and suppresses immune responses after sepsis. PLoS One.

[29] Oberst A (2011). Catalytic activity of the caspase-8-FLIP(L) complex inhibits RIPK3-dependent necrosis. Nature.

[30] Li H (2010). Cellular FLICE-inhibitory protein protects against cardiac remodeling induced by angiotensin II in mice. Hypertension.

[31] Browning JD, Horton JD (2004). Molecular mediators of hepatic steatosis and liver injury. J. Clin. Invest..

[32] Tao L, Zhu Y, Liu J (2023). Identification of new co-diagnostic genes for sepsis and metabolic syndrome using single-cell data analysis and machine learning algorithms. Front. Genet..

[33] Bannerman DD, Eiting KT, Winn RK, Harlan JM (2004). FLICE-like inhibitory protein (FLIP) protects against apoptosis and suppresses NF-kappaB activation induced by bacterial lipopolysaccharide. Am. J. Pathol..

[34] Komarova EA (2005). p53 is a suppressor of inflammatory response in mice. FASEB J..

[35] Guegan JP (2020). CD95/Fas and metastatic disease: What does not kill you makes you stronger. Semin. Cancer Biol..

[36] Ayala A, Lomas JL, Grutkoski PS, Chung CS (2003). Fas-Ligand mediated apoptosis in severe sepsis and shock. Scand. J. Infect. Dis..

[37] Chung CS (2001). Inhibition of Fas signaling prevents hepatic injury and improves organ blood flow during sepsis. Surgery.

[38] Chung CS (2003). Inhibition of Fas/Fas ligand signaling improves septic survival: differential effects on macrophage apoptotic and functional capacity. J. Leukoc. Biol..

[39] Cambier S, Gouwy M, Proost P (2023). The chemokines CXCL8 and CXCL12: Molecular and functional properties, role in disease and efforts towards pharmacological intervention. Cell. Mol. Immunol..

[40] Wang Y (2023). The role of G protein-coupled receptor in neutrophil dysfunction during sepsis-induced acute respiratory distress syndrome. Front. Immunol..

[41] Shen XF, Cao K, Jiang JP, Guan WX, Du JF (2017). Neutrophil dysregulation during sepsis: An overview and update. J. Cell. Mol. Med..

[42] Torgersen C (2009). Macroscopic postmortem findings in 235 surgical intensive care patients with sepsis. Anesth. Analg..

[43] Paudel S (2019). CXCL1 regulates neutrophil homeostasis in pneumonia-derived sepsis caused by Streptococcus pneumoniae serotype 3. Blood.

[44] Spec A (2016). T cells from patients with Candida sepsis display a suppressive immunophenotype. Crit. Care.

[45] Danahy DB, Strother RK, Badovinac VP, Griffith TS (2016). Clinical and experimental sepsis impairs CD8 T-cell-mediated immunity. Crit. Rev. Immunol..

[46] Yang X (2023). Development of cell permeable NanoBRET probes for the measurement of PLK1 target engagement in live cells. Molecules.

[47] Pal-Ghosh R (2021). CDC2 is an important driver of vascular smooth muscle cell proliferation via FOXM1 and PLK1 in pulmonary arterial hypertension. Int. J. Mol. Sci..

[48] Carroll KR (2018). Extending remission and reversing New-onset type 1 diabetes by targeted ablation of autoreactive T cells. Diabetes.

[49] Chen J (2021). Targeting WEE1 by adavosertib inhibits the malignant phenotypes of hepatocellular carcinoma. Biochem Pharmacol.

