# Supplementary material for: A novel anoikis-related gene signature predicts prognosis in patients with sepsis and reveals immune infiltration
Source: Sci Rep. 2024 Jan 28;14:2313. doi: 10.1038/s41598-024-52742-9 (PMC10822872; doi:10.1038/s41598-024-52742-9)
Supplement: Supplementary file 3 — Supplementary Table 1. [file 41598_2024_52742_MOESM3_ESM.docx]

Supplementary Table 1. Anoikis-related genes from GeneCards

| Gene Symbol | GC ID | Gene Symbol | GC ID | Gene Symbol | GC ID |  |
| --- | --- | --- | --- | --- | --- | --- |
| BRMS1 | GC11M116931 | EGF | GC04P109912 | SOD2 | GC06M159669 |  |
| PTK2 | GC08M140657 | ITGB4 | GC17P075721 | MAPK11 | GC22M050263 |  |
| NTRK2 | GC09P084668 | CERNA3 | GC08P056096 | SOD2-OT1 | GC06M159772 |  |
| BCL2L11 | GC02P111119 | DAPK1 | GC09P087497 | PTHLH | GC12M027959 |  |
| SRC | GC20P037344 | MIR145 | GC05P149430 | RIPK1 | GC06P003825 |  |
| CAV1 | GC07P116524 | EZH2 | GC07M148807 | PDGFB | GC22M073492 |  |
| AKT1 | GC14M104769 | PIK3R1 | GC05P068215 | GLI2 | GC02P120735 |  |
| ITGB1 | GC10M035059 | MAP2K1 | GC15P066386 | LINC00958 | GC11M012877 | |
| CEACAM6 | GC19P041750 | CHEK2 | GC22M028687 | CXCR4 | GC02M136114 |  |
| EGFR | GC07P055019 | CXCL12 | GC10M044370 | HMGA1 | GC06P119025 |  |
| CASP8 | GC02P201233 | E2F1 | GC20M033675 | SIK2 | GC11P112292 |  |
| BCL2 | GC18M063123 | LGALS3 | GC14P055124 | TNFSF10 | GC03M172505 |  |
| STAT3 | GC17M042313 | BAK1 | GC06M033572 | NOTCH1 | GC09M138467 |  |
| CEACAM5 | GC19P096057 | ABHD4 | GC14P041737 | ANGPTL2 | GC09M127087 |  |
| TP53 | GC17M007661 | CD44 | GC11P035139 | NTF3 | GC12P033693 |  |
| PTRH2 | GC17M059674 | PIK3R3 | GC01M046041 | S100A4 | GC01M153543 |  |
| SIK1 | GC21M043414 | ITGA4 | GC02P181456 | ETV4 | GC17M043527 |  |
| MAPK1 | GC22M021759 | FADD | GC11P070203 | MIR124-1 | GC08M009903 |  |
| TLE1 | GC09M081583 | PHLDA2 | GC11M002928 | STAT6 | GC12M057095 |  |
| CTNNB1 | GC03P041194 | MIR203A | GC14P113810 | EIF2AK3 | GC02M088556 |  |
| BMF | GC15M040087 | TGFB1 | GC19M041301 | ACLY | GC17M041866 |  |
| DAPK2 | GC15M063907 | HMCN1 | GC01P185734 | LAMC2 | GC01P183186 |  |
| ITGA5 | GC12M055460 | MMP2 | GC16P061423 | LAMB3 | GC01M209614 |  |
| ZNF304 | GC19P057351 | CEBPB | GC20P050190 | PRDX6 | GC01P173477 |  |
| MCL1 | GC01M158584 | CEMIP | GC15P080779 | TRAF6 | GC11M036467 |  |
| BCL2L1 | GC20M031664 | CDKN3 | GC14P054398 | LAMA3 | GC18P023689 |  |
| CASP3 | GC04M184627 | LINC01672 | GC01P008900 | PHB1 | GC17M068585 |  |
| CDH1 | GC16P068737 | CBL | GC11P119206 | PTK7 | GC06P043076 |  |
| BDNF-AS | GC11P027466 | CASP9 | GC01M015491 | KIF2A | GC05P062306 |  |
| BAD | GC11M116809 | SFN | GC01P031745 | RAB1B | GC11P082005 |  |
| MAPK3 | GC16M042426 | MTDH | GC08P097644 | PELP1 | GC17M004669 |  |
| PAK1 | GC11M117322 | PRKCA | GC17P066302 | HTRA1 | GC10P122461 |  |
| ITGAV | GC02P186589 | TNFRSF10B | GC08M023020 | LATS1 | GC06M149658 |  |
| MIR7-3HG | GC19P094936 | CXCL8 | GC04P073740 | CEACAM3 | GC19P041796 |  |
| FN1 | GC02M215360 | MIR200C | GC12P033755 | CDH2 | GC18M032084 |  |
| PIK3CA | GC03P179148 | CDKN2A | GC09M021967 | CSNK2A1 | GC20M000472 |  |
| PTGS2 | GC01M186671 | PIK3CB | GC03M138652 | EDIL3 | GC05M083940 |  |
| BAX | GC19P048954 | CLDN1 | GC03M190305 | ZEB2 | GC02M144384 |  |
| BCAR1 | GC16M075228 | MIR204 | GC09M070809 | TLN1 | GC09M035696 |  |
| TIMP1 | GC0XP053104 | MIR26A1 | GC03P037969 | MIR200B | GC01P001167 |  |
| ERBB2 | GC17P039687 | AR | GC0XP067544 | SIRT3 | GC11M000215 |  |
| PTEN | GC10P104451 | CDKN1A | GC06P119063 | OLFM3 | GC01M101802 |  |
| ANGPTL4 | GC19P008363 | CDKN1B | GC12P033950 | CLU | GC08M027596 |  |
| CYCS | GC07M025118 | KLF12 | GC13M073686 | SPINK1 | GC05M147825 |  |
| BRAF | GC07M140757 | PDGFRB | GC05M150113 | CPEB2 | GC04P023031 |  |
| YAP1 | GC11P102110 | MYC | GC08P127735 | SMAD5-AS1 | GC05M136129 |  |
| ITGA2 | GC05P052989 | SMAD4 | GC18P051028 | NAT1 | GC08P018183 |  |
| ANXA5 | GC04M121667 | PLAU | GC10P073909 | TSG101 | GC11M018468 |  |
| BIRC5 | GC17P078214 | PLK1 | GC16P060461 | MIR200A | GC01P008374 |  |
| ATF4 | GC22P039519 | MUC1 | GC01M155185 | MIR6744 | GC11P001256 |  |
| BDNF | GC11M027654 | LGALS1 | GC22P037675 | SERPINA1 | GC14M094376 |  |
| SCARNA5 | GC02P233275 | PYCARD | GC16M031201 | AKT3 | GC01M243488 |  |
| CSPG4 | GC15M075674 | SESN2 | GC01P031838 | MAPK14 | GC06P119053 |  |
| BSG | GC19P000571 | NTRK1 | GC01P156815 | RELA | GC11M065653 |  |
| AKT2 | GC19M040230 | KRAS | GC12M027012 | RPS6KA1 | GC01P031698 |  |
| MAPK8 | GC10P048306 | ITGB3 | GC17P093864 | TNFRSF1A | GC12M006328 |  |
| IGF1 | GC12M102395 | CCN2 | GC06M131948 | FASLG | GC01P172659 |  |
| IGF1R | GC15P098648 | NRAS | GC01M114704 | AFP | GC04P073431 |  |
| MTOR | GC01M011106 | BID | GC22M017734 | EEF1A1 | GC06M087895 |  |
| FBXW7-AS1 | GC04P152338 | THBS1 | GC15P039581 | ITGA8 | GC10M015513 |  |
| ITGA6 | GC02P172427 | HRAS | GC11M009562 | SATB1 | GC03M025064 |  |
| ANKRD13C | GC01M070259 | CDK11B | GC01M011595 | PBK | GC08M027809 |  |
| ILK | GC11P007159 | CDK11A | GC01M001702 | CD63 | GC12M055727 |  |
| CFLAR | GC02P201413 | CSK | GC15P074782 | LTB4R2 | GC14P041769 |  |
| RHOA | GC03M049359 | PPARG | GC03P012287 | NOX4 | GC11M089324 |  |
| HIF1A | GC14P061695 | IL6 | GC07P022725 | MAVS | GC20P007226 |  |
| DAP3 | GC01P158876 | MYH9 | GC22M036281 | HRC | GC19M049151 |  |
| STK11 | GC19P001177 | CCR7 | GC17M067978 | RHOB | GC02P020447 |  |
| PDK4 | GC07M095583 | MSLN | GC16P059755 | PPP1R13B | GC14M103733 |  |
| XIAP | GC0XP123859 | CCDC178 | GC18M032937 | MET | GC07P116672 |  |
| ITGA3 | GC17P050055 | RAC1 | GC07P009178 | PLG | GC06P160702 |  |
| PTK2B | GC08P027311 | GRHL2 | GC08P101492 | RAF1 | GC03M012583 |  |
| CCND1 | GC11P069641 | BIRC3 | GC11P102317 | EPHA2 | GC01M016124 |  |
| CTTN | GC11P070398 | RHOG | GC11M009723 | PARP1 | GC01M226360 |  |
| CALR | GC19P012938 | CCAR2 | GC08P022604 | BRCA2 | GC13P032315 |  |
| TMX2-CTNND1 | GC11P057712 | NQO1 | GC16M069706 | MAPK10 | GC04M085990 |  |
| CDCP1 | GC03M045082 | MIR21 | GC17P094092 | PRKCQ | GC10M006775 |  |
| TRC-GCA24-1 | GC17M067898 | MMP13 | GC11M102942 | RB1 | GC13P048303 |  |
| CPT1A | GC11M068754 | FAS | GC10P104467 | DOCK1 | GC10P126905 |  |
| PLAUR | GC19M043646 | MTA1 | GC14P105419 | SP1 | GC12P053380 |  |
| SKP2 | GC05P036151 | MYO5A | GC15M142209 | HAVCR2 | GC05M157063 |  |
| MYBBP1A | GC17M004538 | EDA2R | GC0XM066595 | PDCD4 | GC10P110871 |  |
| HGF | GC07M081699 | CCN6 | GC06P120028 | VTN | GC17M067441 |  |
| TLE5 | GC19M016624 | MMP9 | GC20P046008 | INHBB | GC02P128987 |  |
| PIK3CG | GC07P106865 | ABL1 | GC09P130713 |  |  |  |
