# Supplementary material for: A novel anoikis-related gene signature predicts prognosis in patients with sepsis and reveals immune infiltration
Source: Sci Rep. 2024 Jan 28;14:2313. doi: 10.1038/s41598-024-52742-9 (PMC10822872; doi:10.1038/s41598-024-52742-9)
Supplement: Supplementary file 4 — Supplementary Table 2. [file 41598_2024_52742_MOESM4_ESM.docx]

Supplementary Table 2. Predicted miRNAs targeting to prognostic DEARGs by miRNet

| miRNA | mRNA | miRNA | mRNA | miRNA | mRNA |
| --- | --- | --- | --- | --- | --- |
| let-7a-5p | CXCL8 | mir-3165 | TP53 | mir-520c-3p | CFLAR |
| let-7b-5p | CXCL8 | mir-3065-5p | TP53 | mir-616-3p | CFLAR |
| let-7c-5p | CXCL8 | mir-4306 | TP53 | mir-766-3p | CFLAR |
| let-7d-5p | CXCL8 | mir-4316 | CXCL8 | mir-9-5p | CFLAR |
| let-7e-5p | FASLG | mir-4325 | FASLG | mir-99a-5p | CFLAR |
| let-7e-5p | CXCL8 | mir-4271 | TP53 | mir-99b-5p | CFLAR |
| let-7f-5p | CXCL8 | mir-4276 | TP53 | mir-494-3p | CFLAR |
| mir-15a-5p | TP53 | mir-4286 | TP53 | mir-124-3p | CFLAR |
| mir-16-5p | TP53 | mir-3941 | FASLG | mir-126-3p | CFLAR |
| mir-17-5p | TP53 | mir-4419a | TP53 | mir-129-2-3p | CFLAR |
| mir-18a-5p | TP53 | mir-4426 | CXCL8 | mir-191-5p | CFLAR |
| mir-19a-3p | TP53 | mir-4434 | TP53 | mir-19a-3p | CFLAR |
| mir-19b-3p | TP53 | mir-4458 | CXCL8 | mir-27a-3p | FASLG |
| mir-20a-5p | TP53 | mir-4500 | CXCL8 | mir-221-3p | FASLG |
| mir-21-5p | FASLG | mir-4510 | TP53 | let-7a-5p | TP53 |
| mir-23a-3p | CXCL8 | mir-4516 | TP53 | let-7b-5p | TP53 |
| mir-24-3p | TP53 | mir-4531 | TP53 | let-7c-5p | TP53 |
| mir-25-3p | FASLG | mir-4644 | TP53 | let-7d-5p | TP53 |
| mir-25-3p | TP53 | mir-4647 | CXCL8 | let-7e-5p | TP53 |
| mir-26a-5p | CFLAR | mir-4650-5p | TP53 | let-7f-5p | TP53 |
| mir-26b-5p | FASLG | mir-4651 | TP53 | let-7g-5p | TP53 |
| mir-27a-3p | TP53 | mir-4662b | CXCL8 | let-7i-5p | TP53 |
| mir-28-5p | TP53 | mir-4677-3p | CXCL8 | mir-103a-3p | TP53 |
| mir-30a-5p | TP53 | mir-4682 | TP53 | mir-107 | TP53 |
| mir-32-5p | FASLG | mir-4697-3p | TP53 | mir-122-5p | TP53 |
| mir-92a-3p | FASLG | mir-4725-3p | TP53 | mir-1246 | TP53 |
| mir-93-5p | CXCL8 | mir-4728-5p | TP53 | mir-128-3p | TP53 |
| mir-98-5p | CXCL8 | mir-4736 | TP53 | mir-181a-5p | TP53 |
| mir-106a-5p | CXCL8 | mir-499b-3p | CXCL8 | mir-182-5p | TP53 |
| mir-106a-5p | TP53 | mir-4763-3p | TP53 | mir-183-5p | TP53 |
| mir-30c-5p | TP53 | mir-4795-5p | TP53 | mir-188-5p | TP53 |
| mir-30d-5p | TP53 | mir-1273f | TP53 | mir-205-5p | TP53 |
| mir-7-5p | CFLAR | mir-5003-5p | TP53 | mir-21-3p | TP53 |
| mir-10b-5p | TP53 | mir-5193 | TP53 | mir-218-5p | TP53 |
| mir-34a-5p | FASLG | mir-5195-5p | FASLG | mir-22-3p | TP53 |
| mir-34a-5p | TP53 | mir-5196-3p | TP53 | mir-27b-3p | TP53 |
| mir-34a-5p | CFLAR | mir-5197-5p | TP53 | mir-29a-3p | TP53 |
| mir-203a-3p | CXCL8 | mir-5587-3p | TP53 | mir-29b-3p | TP53 |
| mir-204-5p | CXCL8 | mir-5693 | TP53 | mir-29c-3p | TP53 |
| mir-214-3p | TP53 | mir-5702 | TP53 | mir-361-3p | TP53 |
| mir-221-3p | TP53 | mir-5703 | TP53 | mir-362-5p | TP53 |
| mir-222-3p | TP53 | mir-539-3p | FASLG | mir-3657 | TP53 |
| mir-223-3p | TP53 | mir-660-3p | TP53 | mir-500b-5p | TP53 |
| let-7g-5p | CXCL8 | mir-1247-3p | TP53 | mir-522-5p | TP53 |
| let-7i-5p | CXCL8 | mir-3529-3p | TP53 | mir-766-3p | TP53 |
| mir-1-3p | CXCL8 | mir-937-5p | TP53 | mir-93-5p | TP53 |
| mir-30b-5p | TP53 | mir-1233-5p | TP53 | mir-940 | TP53 |
| mir-124-3p | CXCL8 | mir-6127 | TP53 | mir-9-5p | TP53 |
| mir-125b-5p | TP53 | mir-6129 | TP53 | mir-98-5p | TP53 |
| mir-125a-5p | TP53 | mir-6130 | TP53 | mir-1-3p | TP53 |
| mir-146a-5p | CXCL8 | mir-6133 | TP53 | mir-23b-3p | TP53 |
| mir-149-5p | FASLG | mir-6722-3p | TP53 | mir-155-5p | TP53 |
| mir-150-5p | TP53 | mir-1296-3p | TP53 | mir-191-5p | TP53 |
| mir-185-5p | TP53 | mir-513b-3p | TP53 | mir-203a-3p | TP53 |
| mir-155-5p | CXCL8 | mir-6731-5p | TP53 | mir-210-3p | TP53 |
| mir-106b-5p | TP53 | mir-6749-3p | TP53 | mir-212-3p | TP53 |
| mir-200a-3p | TP53 | mir-6751-5p | TP53 | mir-148b-3p | TP53 |
| mir-30e-5p | TP53 | mir-6752-5p | TP53 | mir-106b-5p | CXCL8 |
| mir-363-3p | FASLG | mir-6756-5p | TP53 | mir-10a-3p | CXCL8 |
| mir-302c-3p | CXCL8 | mir-6760-5p | TP53 | mir-1225-5p | CXCL8 |
| mir-302d-3p | CXCL8 | mir-6760-3p | CXCL8 | mir-1291 | CXCL8 |
| mir-367-3p | FASLG | mir-6766-5p | TP53 | mir-138-5p | CXCL8 |
| mir-375 | TP53 | mir-6778-5p | TP53 | mir-17-5p | CXCL8 |
| mir-377-3p | TP53 | mir-6785-5p | TP53 | mir-195-5p | CXCL8 |
| mir-330-3p | TP53 | mir-6797-5p | TP53 | mir-20a-5p | CXCL8 |
| mir-324-5p | TP53 | mir-6803-5p | TP53 | mir-20b-5p | CXCL8 |
| mir-335-5p | CXCL8 | mir-6825-5p | TP53 | mir-23b-3p | CXCL8 |
| mir-346 | CFLAR | mir-6835-5p | TP53 | mir-296-3p | CXCL8 |
| mir-329-3p | FASLG | mir-6780b-5p | TP53 | mir-302c-5p | CXCL8 |
| mir-485-5p | TP53 | mir-6842-5p | TP53 | mir-302d-5p | CXCL8 |
| mir-485-3p | FASLG | mir-6880-5p | TP53 | mir-30c-1-3p | CXCL8 |
| mir-491-5p | TP53 | mir-6882-5p | TP53 | mir-34a-5p | CXCL8 |
| mir-202-3p | CXCL8 | mir-6883-5p | TP53 | mir-373-3p | CXCL8 |
| mir-520b | CXCL8 | mir-7110-5p | TP53 | mir-450a-5p | CXCL8 |
| mir-518c-3p | TP53 | mir-7150 | TP53 | mir-493-5p | CXCL8 |
| mir-504-5p | TP53 | mir-7703 | FASLG | mir-519d-3p | CXCL8 |
| mir-92b-3p | FASLG | mir-7849-3p | FASLG | mir-520a-3p | CXCL8 |
| mir-603 | FASLG | mir-8071 | TP53 | mir-520c-3p | CXCL8 |
| mir-605-5p | TP53 | mir-8085 | TP53 | mir-526b-3p | CXCL8 |
| mir-608 | TP53 | mir-1249-5p | TP53 | mir-5582-3p | CXCL8 |
| mir-612 | TP53 | mir-8485 | FASLG | mir-587 | CXCL8 |
| mir-622 | TP53 | mir-100-5p | CFLAR | mir-664a-3p | CXCL8 |
| mir-638 | TP53 | mir-101-5p | CFLAR | mir-101-3p | CXCL8 |
| mir-663a | TP53 | mir-103a-3p | CFLAR | mir-107 | CXCL8 |
| mir-454-3p | TP53 | mir-107 | CFLAR | mir-126-3p | CXCL8 |
| mir-21-3p | FASLG | mir-122-5p | CFLAR | mir-129-2-3p | CXCL8 |
| mir-26a-1-3p | TP53 | mir-1246 | CFLAR | mir-130a-3p | CXCL8 |
| mir-28-3p | TP53 | mir-125a-5p | CFLAR | mir-147a | CXCL8 |
| mir-92a-1-5p | TP53 | mir-125b-5p | CFLAR | mir-16-5p | CXCL8 |
| mir-92a-2-5p | TP53 | mir-1270 | CFLAR | mir-194-5p | CXCL8 |
| mir-100-3p | CXCL8 | mir-1303 | CFLAR | mir-21-3p | CXCL8 |
| mir-214-5p | TP53 | mir-132-3p | CFLAR | mir-21-5p | CXCL8 |
| mir-125b-1-3p | TP53 | mir-135a-5p | CFLAR | mir-210-3p | CXCL8 |
| mir-149-3p | TP53 | mir-135b-5p | CFLAR | mir-212-3p | CXCL8 |
| mir-150-3p | TP53 | mir-142-3p | CFLAR | mir-214-3p | CXCL8 |
| mir-155-3p | TP53 | mir-155-5p | CFLAR | mir-221-3p | CXCL8 |
| mir-194-3p | TP53 | mir-15a-5p | CFLAR | mir-27a-3p | CXCL8 |
| mir-362-3p | FASLG | mir-15b-5p | CFLAR | mir-27a-5p | CXCL8 |
| mir-151a-5p | TP53 | mir-16-5p | CFLAR | mir-29a-3p | CXCL8 |
| mir-499a-3p | CXCL8 | mir-195-5p | CFLAR | mir-30d-5p | CXCL8 |
| mir-505-5p | TP53 | mir-2115-5p | CFLAR | mir-376a-5p | CXCL8 |
| mir-629-5p | CFLAR | mir-212-3p | CFLAR | mir-429 | CXCL8 |
| mir-300 | TP53 | mir-22-5p | CFLAR | mir-671-5p | CXCL8 |
| mir-1225-3p | TP53 | mir-3199 | CFLAR | mir-7-5p | CXCL8 |
| mir-1228-3p | TP53 | mir-324-3p | CFLAR | mir-941 | CXCL8 |
| mir-1264 | TP53 | mir-326 | CFLAR | mir-99b-5p | CXCL8 |
| mir-1207-5p | TP53 | mir-330-5p | CFLAR | mir-520f-3p | CXCL8 |
| mir-1208 | CXCL8 | mir-3613-3p | CFLAR | mir-372-3p | CXCL8 |
| mir-1285-3p | TP53 | mir-4326 | CFLAR | mir-302a-3p | CXCL8 |
| mir-1294 | CXCL8 | mir-484 | CFLAR | mir-148b-3p | CXCL8 |
| mir-1909-3p | TP53 | mir-491-5p | CFLAR | mir-133a-3p | CXCL8 |
| mir-1972 | TP53 | mir-509-3p | CFLAR | mir-9-5p | CXCL8 |
| mir-2110 | TP53 | mir-520a-3p | CFLAR | mir-30a-5p | CXCL8 |
| mir-1285 | TP53 | mir-512-3p | CFLAR |  |  |
