# Supplementary material for: A novel anoikis-related gene signature predicts prognosis in patients with sepsis and reveals immune infiltration
Source: Sci Rep. 2024 Jan 28;14:2313. doi: 10.1038/s41598-024-52742-9 (PMC10822872; doi:10.1038/s41598-024-52742-9)
Supplement: Supplementary file 5 — Supplementary Table 3. [file 41598_2024_52742_MOESM5_ESM.docx]

Supplementary Table 3. Differentially expressed miRNAs between septic patients and healthy controls in GSE134358

| miRNA | Log FC | P value | miRNA | Log FC | P value |
| --- | --- | --- | --- | --- | --- |
| ipu-miR-150 | -2.61145 | 1.02E-36 | tgu-miR-106-5p | -1.20746 | 1.04E-07 |
| mdo-miR-150-5p | -2.21303 | 4.00E-28 | mdo-miR-15a-5p | -1.20242 | 7.36E-06 |
| oan-miR-150-5p | -2.14021 | 8.06E-27 | hsa-miR-6877-5p | -1.19856 | 5.91E-19 |
| aca-miR-150-5p | -2.10916 | 5.17E-34 | tgu-miR-29b-2-5p | -1.19638 | 3.25E-08 |
| cfa-miR-150 | -2.10916 | 5.17E-34 | cfa-miR-106a | -1.1906 | 1.54E-07 |
| eca-miR-150 | -2.10916 | 5.17E-34 | bta-miR-494 | -1.18806 | 2.15E-07 |
| ggo-miR-150 | -2.10916 | 5.17E-34 | cfa-miR-494 | -1.18806 | 2.15E-07 |
| mml-miR-150-5p | -2.10916 | 5.17E-34 | eca-miR-494 | -1.18806 | 2.15E-07 |
| oar-miR-150 | -2.10916 | 5.17E-34 | ggo-miR-494 | -1.18806 | 2.15E-07 |
| ppy-miR-150 | -2.10916 | 5.17E-34 | hsa-miR-494-3p | -1.18806 | 2.15E-07 |
| ptr-miR-150 | -2.10916 | 5.17E-34 | mml-miR-494-3p | -1.18806 | 2.15E-07 |
| ssc-miR-150 | -2.10916 | 5.17E-34 | ppy-miR-494 | -1.18806 | 2.15E-07 |
| tgu-miR-142-5p | -2.1002 | 1.30E-19 | ptr-miR-494 | -1.18806 | 2.15E-07 |
| aca-miR-126-3p | -1.99513 | 4.67E-12 | aca-miR-17-5p | -1.1736 | 8.91E-07 |
| cgr-miR-126 | -1.99513 | 4.67E-12 | ccr-miR-17-5p | -1.1736 | 8.91E-07 |
| eca-miR-126-3p | -1.99513 | 4.67E-12 | cgr-miR-17-5p | -1.1736 | 8.91E-07 |
| hsa-miR-126-3p | -1.99513 | 4.67E-12 | eca-miR-106a | -1.1736 | 8.91E-07 |
| mdo-miR-126-3p | -1.99513 | 4.67E-12 | eca-miR-17 | -1.1736 | 8.91E-07 |
| mml-miR-126 | -1.99513 | 4.67E-12 | oan-miR-17-5p | -1.1736 | 8.91E-07 |
| oan-miR-126-3p | -1.99513 | 4.67E-12 | rno-miR-17-5p | -1.1736 | 8.91E-07 |
| ppy-miR-126 | -1.99513 | 4.67E-12 | ssc-miR-17-5p | -1.1736 | 8.91E-07 |
| ptr-miR-126 | -1.99513 | 4.67E-12 | tgu-miR-17a-5p | -1.1736 | 8.91E-07 |
| ssc-miR-126-3p | -1.99513 | 4.67E-12 | sbi-miR6224a-3p | -1.17187 | 1.29E-05 |
| tgu-miR-126-3p | -1.99513 | 4.67E-12 | sbi-miR6224b-3p | -1.17187 | 1.29E-05 |
| aca-miR-20a-5p | -1.92172 | 1.21E-11 | sbi-miR6224c-3p | -1.17187 | 1.29E-05 |
| age-miR-20 | -1.92172 | 1.21E-11 | cgr-miR-15a-5p | -1.16761 | 1.21E-06 |
| ggo-miR-20a | -1.92172 | 1.21E-11 | cin-let-7a-5p | -1.16509 | 1.11E-13 |
| lca-miR-20 | -1.92172 | 1.21E-11 | aca-miR-20b-5p | -1.15668 | 1.76E-06 |
| lla-miR-20 | -1.92172 | 1.21E-11 | age-miR-106a | -1.15389 | 2.91E-07 |
| mne-miR-20 | -1.92172 | 1.21E-11 | ggo-miR-106a | -1.15389 | 2.91E-07 |
| ppa-miR-20 | -1.92172 | 1.21E-11 | mne-miR-106a | -1.15389 | 2.91E-07 |
| ppy-miR-20a | -1.92172 | 1.21E-11 | ppa-miR-106a | -1.15389 | 2.91E-07 |
| ptr-miR-20a | -1.92172 | 1.21E-11 | ppy-miR-106a | -1.15389 | 2.91E-07 |
| sla-miR-20 | -1.92172 | 1.21E-11 | ptr-miR-106a | -1.15389 | 2.91E-07 |
| ssc-miR-20a | -1.92172 | 1.21E-11 | sla-miR-106a | -1.15389 | 2.91E-07 |
| ola-miR-20a | -1.88394 | 4.58E-12 | ssc-miR-106a | -1.15389 | 2.91E-07 |
| cfa-miR-142 | -1.88285 | 3.80E-18 | gga-let-7d | -1.15378 | 2.21E-06 |
| ggo-miR-142 | -1.88285 | 3.80E-18 | ccr-miR-19d | -1.15254 | 5.81E-05 |
| sha-miR-142 | -1.88285 | 3.80E-18 | age-miR-17-5p | -1.14751 | 5.11E-07 |
| mdo-miR-106-5p | -1.87432 | 1.66E-11 | ggo-miR-17-5p | -1.14751 | 5.11E-07 |
| cfa-miR-20b | -1.86992 | 1.13E-10 | lca-miR-17-5p | -1.14751 | 5.11E-07 |
| ccr-miR-20a-5p | -1.84283 | 2.62E-11 | lla-miR-17-5p | -1.14751 | 5.11E-07 |
| cfa-miR-20a | -1.84283 | 2.62E-11 | mne-miR-17-5p | -1.14751 | 5.11E-07 |
| cgr-miR-20a | -1.84283 | 2.62E-11 | ppa-miR-17-5p | -1.14751 | 5.11E-07 |
| eca-miR-20a | -1.84283 | 2.62E-11 | ppy-miR-17-5p | -1.14751 | 5.11E-07 |
| tgu-miR-20a-5p | -1.84283 | 2.62E-11 | ptr-miR-17-5p | -1.14751 | 5.11E-07 |
| xtr-miR-20a-5p | -1.84283 | 2.62E-11 | sla-miR-17-5p | -1.14751 | 5.11E-07 |
| pma-let-7b-5p | -1.83934 | 9.13E-17 | ccr-let-7i | -1.14464 | 1.64E-09 |
| aca-miR-18a-5p | -1.77811 | 1.13E-10 | gga-let-7i | -1.14464 | 1.64E-09 |
| ccr-miR-18a | -1.77811 | 1.13E-10 | ggo-let-7i | -1.14464 | 1.64E-09 |
| cgr-miR-18a-5p | -1.77811 | 1.13E-10 | sha-let-7i | -1.14464 | 1.64E-09 |
| eca-miR-18a | -1.77811 | 1.13E-10 | aca-miR-1306 | -1.13923 | 4.76E-18 |
| ola-miR-18 | -1.77811 | 1.13E-10 | cre-miR1142 | -1.1313 | 1.53E-08 |
| pma-miR-18a-5p | -1.77811 | 1.13E-10 | aca-let-7i-5p | -1.12855 | 1.07E-09 |
| rno-miR-18a-5p | -1.77811 | 1.13E-10 | ipu-let-7i | -1.12855 | 1.07E-09 |
| xtr-miR-18a-5p | -1.77811 | 1.13E-10 | mml-let-7i-5p | -1.12855 | 1.07E-09 |
| hsa-miR-548q | -1.75562 | 7.90E-26 | oar-let-7i | -1.12855 | 1.07E-09 |
| pma-miR-20a-5p | -1.74868 | 2.23E-10 | ppy-let-7i | -1.12855 | 1.07E-09 |
| gga-miR-126-3p | -1.73639 | 9.46E-11 | ptr-let-7i | -1.12855 | 1.07E-09 |
| cgr-miR-142-5p | -1.73062 | 4.16E-11 | tgu-let-7i-5p | -1.12855 | 1.07E-09 |
| gga-miR-142-5p | -1.73062 | 4.16E-11 | ccr-miR-16b | -1.12388 | 3.86E-06 |
| csa-let-7c | -1.62894 | 2.96E-14 | ipu-miR-16b | -1.12388 | 3.86E-06 |
| age-miR-15a | -1.60686 | 1.20E-08 | tgu-miR-16b-5p | -1.12388 | 3.86E-06 |
| eca-miR-15a | -1.60686 | 1.20E-08 | eca-miR-20b | -1.11952 | 4.70E-06 |
| ggo-miR-15a | -1.60686 | 1.20E-08 | ggo-miR-20b | -1.11952 | 4.70E-06 |
| lca-miR-15a | -1.60686 | 1.20E-08 | mdo-miR-20b-5p | -1.11952 | 4.70E-06 |
| lla-miR-15a | -1.60686 | 1.20E-08 | mml-miR-20b-5p | -1.11952 | 4.70E-06 |
| mne-miR-15a | -1.60686 | 1.20E-08 | oan-miR-20b-5p | -1.11952 | 4.70E-06 |
| ppa-miR-15a | -1.60686 | 1.20E-08 | ppy-miR-20b | -1.11952 | 4.70E-06 |
| ppy-miR-15a | -1.60686 | 1.20E-08 | ptr-miR-20b | -1.11952 | 4.70E-06 |
| ptr-miR-15a | -1.60686 | 1.20E-08 | tgu-miR-20b-5p | -1.11952 | 4.70E-06 |
| sla-miR-15a | -1.60686 | 1.20E-08 | xla-miR-20 | -1.11952 | 4.70E-06 |
| oar-miR-494-3p | -1.58856 | 2.31E-09 | hsa-miR-6894-3p | -1.11871 | 4.04E-10 |
| age-miR-18 | -1.55478 | 2.12E-09 | ggo-miR-342 | -1.11778 | 5.22E-13 |
| cfa-miR-18a | -1.55478 | 2.12E-09 | xtr-miR-16b | -1.1139 | 1.20E-05 |
| fru-miR-18 | -1.55478 | 2.12E-09 | spu-let-7 | -1.10627 | 9.73E-05 |
| gga-miR-18a-5p | -1.55478 | 2.12E-09 | mmu-miR-7672-5p | -1.08212 | 6.75E-08 |
| ggo-miR-18a | -1.55478 | 2.12E-09 | aca-let-7f-5p | -1.08042 | 0.000143 |
| lca-miR-18 | -1.55478 | 2.12E-09 | bta-let-7f | -1.08042 | 0.000143 |
| lla-miR-18 | -1.55478 | 2.12E-09 | cfa-let-7f | -1.08042 | 0.000143 |
| mne-miR-18 | -1.55478 | 2.12E-09 | cgr-let-7f | -1.08042 | 0.000143 |
| ppa-miR-18 | -1.55478 | 2.12E-09 | eca-let-7f | -1.08042 | 0.000143 |
| ppy-miR-18a | -1.55478 | 2.12E-09 | ipu-let-7f | -1.08042 | 0.000143 |
| ptr-miR-18a | -1.55478 | 2.12E-09 | mml-let-7f-5p | -1.08042 | 0.000143 |
| sla-miR-18 | -1.55478 | 2.12E-09 | oan-let-7f-5p | -1.08042 | 0.000143 |
| ssc-miR-18a | -1.55478 | 2.12E-09 | ppy-let-7f | -1.08042 | 0.000143 |
| tgu-miR-18a | -1.55478 | 2.12E-09 | ptr-let-7f | -1.08042 | 0.000143 |
| ssc-let-7i | -1.48899 | 9.05E-12 | ssc-let-7f | -1.08042 | 0.000143 |
| pma-miR-16-5p | -1.46845 | 4.56E-07 | tgu-let-7f-5p | -1.08042 | 0.000143 |
| cin-let-7b-5p | -1.46825 | 6.55E-17 | xtr-let-7f | -1.08042 | 0.000143 |
| csa-let-7b | -1.46825 | 6.55E-17 | ggo-miR-125a | -1.07562 | 2.41E-05 |
| cfa-miR-15a | -1.45889 | 9.51E-07 | hsa-miR-1270 | -1.07366 | 6.30E-08 |
| gga-miR-15a | -1.45889 | 9.51E-07 | hsa-miR-4539 | -1.0716 | 9.72E-12 |
| oan-miR-15a-5p | -1.45889 | 9.51E-07 | fru-let-7d | -1.06606 | 1.98E-14 |
| ssc-miR-15a | -1.45889 | 9.51E-07 | ipu-let-7d | -1.06606 | 1.98E-14 |
| tgu-miR-15a-5p | -1.45889 | 9.51E-07 | pol-let-7d-5p | -1.06606 | 1.98E-14 |
| pma-let-7c | -1.41444 | 1.69E-10 | bhv1-miR-B10 | -1.05667 | 7.02E-10 |
| gga-miR-1306-3p | -1.40135 | 4.39E-21 | hsa-miR-1255b-5p | -1.05655 | 1.56E-06 |
| ola-miR-106a | -1.38077 | 1.76E-09 | ptr-miR-1255b | -1.05655 | 1.56E-06 |
| mml-miR-628-3p | -1.36353 | 1.13E-10 | cfa-miR-146a | -1.05325 | 0.000421 |
| ppy-miR-628-3p | -1.36353 | 1.13E-10 | cgr-miR-146a | -1.05325 | 0.000421 |
| ptr-miR-628 | -1.36353 | 1.13E-10 | eca-miR-146a | -1.05325 | 0.000421 |
| oan-miR-106-5p | -1.35711 | 6.83E-09 | gga-miR-146a-5p | -1.05325 | 0.000421 |
| oar-miR-106a | -1.35711 | 6.83E-09 | mdo-miR-146a-5p | -1.05325 | 0.000421 |
| ssc-miR-20b | -1.34277 | 3.42E-08 | mml-miR-146a-5p | -1.05325 | 0.000421 |
| pma-miR-20b | -1.29675 | 3.11E-08 | ppy-miR-146a | -1.05325 | 0.000421 |
| aca-miR-16a-5p | -1.29209 | 2.56E-06 | ptr-miR-146a | -1.05325 | 0.000421 |
| oar-miR-16b | -1.29209 | 2.56E-06 | ssc-miR-146a-5p | -1.05325 | 0.000421 |
| ppy-miR-16 | -1.29209 | 2.56E-06 | tgu-miR-146c | -1.05325 | 0.000421 |
| ggo-let-7g | -1.28837 | 2.36E-07 | hsa-miR-500b-3p | -1.05205 | 1.26E-05 |
| mdo-let-7g-5p | -1.28837 | 2.36E-07 | pma-let-7d | -1.05023 | 4.35E-07 |
| oar-let-7g | -1.28837 | 2.36E-07 | lja-miR7539 | -1.04925 | 6.28E-07 |
| sha-let-7g | -1.28837 | 2.36E-07 | hsa-miR-664a-5p | -1.04322 | 3.10E-11 |
| ola-miR-19d | -1.27605 | 4.58E-05 | tgu-miR-130b-3p | -1.03443 | 4.82E-05 |
| eca-miR-485-3p | -1.27571 | 1.10E-07 | cfa-miR-1844 | -1.03149 | 1.61E-08 |
| hsa-miR-485-3p | -1.27571 | 1.10E-07 | hsa-miR-4323 | -1.03025 | 1.84E-09 |
| mml-miR-485-3p | -1.27571 | 1.10E-07 | mmu-miR-1971 | -1.01389 | 2.45E-24 |
| oar-miR-485-3p | -1.27571 | 1.10E-07 | bfl-miR-4898 | -1.0106 | 6.18E-07 |
| ppy-miR-485-3p | -1.27571 | 1.10E-07 | bta-miR-2288 | -1.01052 | 4.64E-09 |
| ptr-miR-485 | -1.27571 | 1.10E-07 | cfa-miR-342 | -1.00931 | 1.81E-14 |
| ccr-miR-93 | -1.27393 | 2.24E-07 | eca-miR-342-3p | -1.00931 | 1.81E-14 |
| sha-miR-19b | -1.27105 | 6.19E-05 | mml-miR-342-3p | -1.00931 | 1.81E-14 |
| ggo-let-7f | -1.26878 | 4.18E-07 | ppy-miR-342-3p | -1.00931 | 1.81E-14 |
| oar-let-7f | -1.26878 | 4.18E-07 | ptr-miR-342 | -1.00931 | 1.81E-14 |
| ggo-let-7e | -1.26777 | 2.97E-13 | mml-miR-500b-3p | -1.00877 | 3.22E-05 |
| cfa-let-7e | -1.24782 | 2.82E-11 | mml-miR-501-3p | -1.00877 | 3.22E-05 |
| eca-let-7e | -1.24782 | 2.82E-11 | gga-miR-6557-5p | 2.028422 | 5.77E-20 |
| mml-let-7e-5p | -1.24782 | 2.82E-11 | hsa-miR-4532 | 2.007724 | 4.88E-26 |
| ppy-let-7e | -1.24782 | 2.82E-11 | hbv-miR-B8-5p | 1.548853 | 5.94E-15 |
| ptr-let-7e | -1.24782 | 2.82E-11 | hsa-miR-7977 | 1.496228 | 1.13E-26 |
| ssc-let-7e | -1.24782 | 2.82E-11 | gga-miR-6596-5p | 1.477304 | 1.14E-09 |
| aca-let-7g | -1.24757 | 3.87E-07 | ipu-miR-7550 | 1.312135 | 1.31E-13 |
| cfa-let-7g | -1.24757 | 3.87E-07 | mse-miR-2779 | 1.312135 | 1.31E-13 |
| cgr-let-7g-5p | -1.24757 | 3.87E-07 | bmo-miR-2779 | 1.306305 | 7.32E-15 |
| eca-let-7g | -1.24757 | 3.87E-07 | bta-miR-2478 | 1.278993 | 9.15E-09 |
| mml-let-7g-5p | -1.24757 | 3.87E-07 | osa-miR5077 | 1.199727 | 8.11E-07 |
| oan-let-7g-5p | -1.24757 | 3.87E-07 | dre-miR-27a | 1.194967 | 1.89E-05 |
| ppy-let-7g | -1.24757 | 3.87E-07 | ipu-miR-27a | 1.194967 | 1.89E-05 |
| ptr-let-7g | -1.24757 | 3.87E-07 | aca-miR-27a-3p | 1.18591 | 0.00013 |
| ssc-let-7g | -1.24757 | 3.87E-07 | ccr-miR-27a | 1.18591 | 0.00013 |
| tgu-let-7g-5p | -1.24757 | 3.87E-07 | cgr-miR-27a-3p | 1.18591 | 0.00013 |
| aca-miR-5452-3p | -1.23913 | 2.02E-07 | eca-miR-27a | 1.18591 | 0.00013 |
| aca-miR-19b | -1.23866 | 2.83E-05 | oan-miR-27a-3p | 1.18591 | 0.00013 |
| hsa-miR-1275 | -1.23784 | 3.36E-25 | oar-miR-27a | 1.18591 | 0.00013 |
| ppy-miR-1275 | -1.23784 | 3.36E-25 | ssc-miR-27a | 1.18591 | 0.00013 |
| ptr-miR-1275 | -1.23784 | 3.36E-25 | hsa-miR-589-5p | 1.120083 | 2.44E-10 |
| ccr-miR-125c | -1.23581 | 2.27E-06 | mml-miR-589 | 1.120083 | 2.44E-10 |
| ipu-miR-125c | -1.23581 | 2.27E-06 | ppy-miR-589 | 1.120083 | 2.44E-10 |
| cgr-miR-1306-3p | -1.23431 | 8.31E-18 | ppt-miR894 | 1.078851 | 1.03E-13 |
| ggo-miR-1306 | -1.23431 | 8.31E-18 | age-miR-27a | 1.054815 | 4.45E-05 |
| mmu-miR-1306-3p | -1.23431 | 8.31E-18 | ggo-miR-27a | 1.054815 | 4.45E-05 |
| ssc-miR-1306-3p | -1.23431 | 8.31E-18 | lca-miR-27a | 1.054815 | 4.45E-05 |
| oar-miR-17-5p | -1.23322 | 6.65E-08 | mne-miR-27a | 1.054815 | 4.45E-05 |
| bta-miR-16a | -1.21879 | 2.13E-06 | ppa-miR-27a | 1.054815 | 4.45E-05 |
| ccr-miR-16a | -1.21879 | 2.13E-06 | ppy-miR-27a | 1.054815 | 4.45E-05 |
| gga-miR-16-5p | -1.21879 | 2.13E-06 | ptr-miR-27a | 1.054815 | 4.45E-05 |
| lca-miR-16 | -1.21879 | 2.13E-06 | sla-miR-27a | 1.054815 | 4.45E-05 |
| oan-miR-16b-5p | -1.21879 | 2.13E-06 | cfa-miR-504 | 1.004924 | 1.42E-06 |
| tgu-miR-16a-5p | -1.21879 | 2.13E-06 | eca-miR-504 | 1.004924 | 1.42E-06 |
| xtr-miR-16a | -1.21879 | 2.13E-06 | mml-miR-504-5p | 1.004924 | 1.42E-06 |
| hsa-miR-942-3p | -1.21256 | 2.72E-09 | ppy-miR-504 | 1.004924 | 1.42E-06 |
| rno-miR-1306-3p | -1.20988 | 2.92E-14 | ptr-miR-504 | 1.004924 | 1.42E-06 |
